# Excited-State Densities from Time-Dependent Density Functional Response Theory

**DOI:** 10.1021/acs.jctc.5c00909

**Published:** 2025-10-10

**Authors:** Anna Baranova, Neepa T. Maitra

**Affiliations:** Department of Physics, 67206 Rutgers University, Newark, New Jersey 07102, United States

## Abstract

While the variational principle for excited-state energies leads to a route to obtaining excited-state densities from time-dependent density functional theory, relatively little attention has been paid to the quality of the resulting densities in real space obtained with different exchange-correlation functional approximations or how nonadiabatic approximations developed for energies of states of double-excitation character perform for their densities. Here we derive an expression directly in real space for the excited-state density, which includes the case of nonadiabatic kernels and consequently is able, for the first time, to yield densities of states of double-excitation character. Under some well-defined simplifications, we compare the performance of the local-density approximation and exact-exchange approximation, which are in a sense at the opposite extremes of the fundamental functional approximations, on local and charge-transfer excitations in one-dimensional model systems and show that the dressed Time-Dependent Density Functional Theory (TDDFT) approach gives good densities of double excitations.

## Graphical abstract

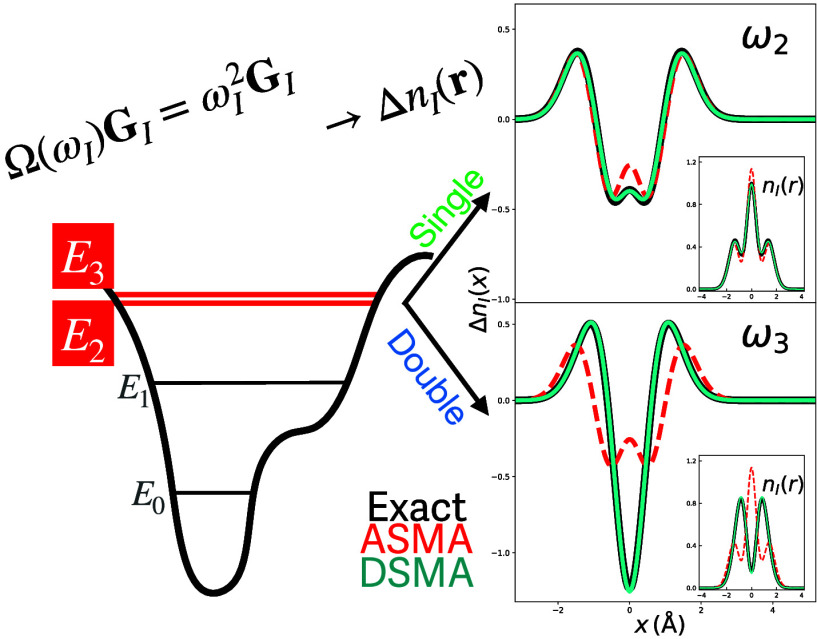

## 1 Introduction

The advent of time-dependent density functional theory (TDDFT) 40 years ago^1–3^ has enabled the calculation of electronic spectra, response properties, and general nonperturbative dynamics, for large and complex systems that would otherwise not be possible. This is due to the one-to-one mapping between time-dependent potentials and densities, for a specified initial state, opening the possibility of finding a noninteracting (Kohn–Sham) system that reproduces the same density while evolving in the computationally simpler one-body Schrödinger equation. While the approach would formally yield exact observables of the interacting system, in practice approximations must be made for the exchange-correlation potential. The majority of applications operate in the linear response regime, where one considers a time-dependent perturbation on the system, from whose response excitation energies and transition properties out of the ground-state can be extracted, and, with the approximations in use today, an unprecedented balance between accuracy and efficiency has been achieved. When thinking about the linear response of a ground-state system, it can be understood that properties related to transition densities between the ground Ψ_0_ and excited states Ψ_*I*_ of the true interacting system, ⟨Ψ_*I*_|*n̂*(**r**)|Ψ_0_⟩, can be directly obtained, *n̂*(**r**) being the density operator, but it is not immediately obvious that excited-state densities ⟨Ψ_*I*_|*n̂*(**r**)|Ψ_*I*_⟩ can be extracted from the formalism. However, with the recognition that excited states are stationary points of the Hamiltonian, then using time-independent perturbation theory instead of time-dependent perturbation theory offers a route to extracting their densities from energies computed from linear response; equivalently, framing the problem as a variational one.^4,5^ (We note that excited-state densities can also be obtained from extrema of the purely the ground-state energy functional,^6,7^ generalized adiabatic connection formalism,^8,9^ stationary principles based on a bifunctional of the excited- and ground-state density,^10,11^ and ensemble DFT approaches^12–15^ ).

In this paper, we work directly in real space instead of in the space of single excitations that the earlier pioneering work was framed in,^4,5^ and go beyond the use of an adiabatic kernel that the earlier work was limited to. The latter extension enables us to obtain densities for states of double-excitation character. We explore the small-matrix approximation limit of our expressions on a series of one-dimensional model systems that cover local excitations, charge-transfer excitations, and double excitations. Motivated by the works^16–23^ suggesting that the hybrid exchange-correlation functionals with a large fraction of exact exchange yield accurate excited-state properties, we explore the performance of the excited-state densities in two opposite limits of approximation–in the adiabatic local-density approximation (LDA) and adiabatic exact exchange (EXX). We find that the choice of ground-state approximation is important in getting good Kohn–Sham densities in the first place, on top of which TDDFT provides corrections that bring the density close to the exact. These corrections involve a sum over all orbitals, whose accuracy depends on the choice of exchange-correlation functional, and we study the convergence of this sum. Finally, we show that the dressed small-matrix (DSMA) and dressed single-pole (DSPA) approximations for frequency-dependent kernel provide good approximations to the densities of states of double-excitation character, while the adiabatic approximation fails.

## 2 Excited-State Densities from Time-Independent Perturbation Theory

A standard result from time-independent perturbation theory of quantum mechanics is that the first-order change to an excited-state energy δ*E*_*I*_ is the expectation value of the perturbation evaluated in that excited state. Applied to many-electron systems when this perturbation is in the externally applied one-body potential *v*_ext_(**r**), this then yields δ*E*_*I*_ = ∫*n*_*I*_(**r**)­δ*v*_ext_(**r**)­d^3^ *r*, where

$$n_{I}\left(r\right)=\sum_{\sigma_{1}\cdot \cdot \cdot \sigma_{N}}\int {\left|\Psi_{I}(r\sigma_{1},r_{2}\sigma_{2\cdot \cdot \cdot }r_{N}\sigma_{N})\right|}^{2}d^{3}r_{2\cdot \cdot \cdot }d^{3}r_{N}$$

is the density of the unperturbed excited state Ψ_*I*_. Thus, knowledge of the excited-state energy and how it varies with the potential yields the excited-state density itself

1 $$n_{I}\left(r\right)=\frac{\delta E_{I}}{\delta v_{\mathrm{ext}}\left(r\right)}$$

Equation 1 provides the cornerstone for obtaining excited-state densities from TDDFT response: while ground-state DFT provides the ground-state energy *E*_0_, the TDDFT linear response formalism provides excitation energies ω_*I*_, so taking the functional derivative with respect to the external potential of the sum of these yields the excited-state density, exact in principle and dependent on functional approximations made in practice, as we shall see shortly. We note that refs 24,25 recently used eq 1 to find the exact excited-state densities of an asymmetric Hubbard dimer from an exact state-dependent excited-state energy functional. Here instead we obtain the energy from TDDFT for general real-space systems. Thus, we write the excited-state energy in two parts

2 $$E_{I}=E_{0}+\omega_{I}$$

where *E*_0_ = ⟨Ψ_0_|*T̂* + *Ŵ*|Ψ_0_⟩ + ∫d^3^ *r* *n*_0_(**r**)*v*_ext_(**r**) (with *T̂* and *Ŵ* as kinetic and electron-interaction operators, Ψ_0_ the ground-state wave function, and *n*_0_ the ground-state density), and ω_*I*_ = *E*_*I*_ – *E*_0_ is the excitation frequency of the excited state *I*. The latter are eigenvalues of the TDDFT matrix equation^26–29^

3 $$\Omega \left(\omega_{I}\right)G_{I}=\omega_{I}^{2}G_{I}$$

where the matrix elements of Ω­(ω) are expressed in the single-excitation basis, *q* = *i* → *a*, *q*′ = *j* → *b*, for spin-saturated systems as

4 $$\Omega_{\mathrm{qq}\prime }\left(\omega_{I}\right)=\nu_{q}^{2}\delta_{\mathrm{qq}\prime }+4\sqrt{\nu_{q}\nu_{q\prime }}f_{\mathrm{HXC},\mathrm{qq}\prime }\left[n_{0}\right]\left(\omega_{I}\right)$$

Here ν_*q*_ = ϵ_*a*_ – ϵ_*i*_, ν_*q*′_ = ϵ_*b*_ – ϵ_*j*_ are the corresponding KS transition frequencies, and *f*_HXC,*qq*′_ is Hartree-exchange-correlation matrix element, defined through

5 $$f_{\mathrm{HXC},\mathrm{qq}\prime }\left[n_{0}\right]\left(\omega_{I}\right)=\iint d^{3}xd^{3}x^{\prime }\Phi_{q}\left(x\right)f_{\mathrm{HXC}}\left[n_{0}\right]\left(x,x^{\prime },\omega_{I}\right)\Phi_{q\prime }\left(x^{\prime }\right)$$

where

6 $$f_{\mathrm{HXC}}\left[n_{0}\right]\left(x,x^{\prime },\omega \right)=\frac{1}{\left|x-x^{\prime }\right|}+\int d\left(t-t^{\prime }\right)e^{i\omega \left(t-t\prime \right)}\frac{\delta v_{\mathrm{XC}}\left(x,t\right)}{\delta n\left(x^{\prime },t^{\prime }\right)}{|}_{n=n_{0}}$$

and Φ_*q*_(**x**) = ϕ_*i*_(**x**)­ϕ_*a*_(**x**) represents a KS single-excitation transition density. We note that while eq 4 represents a matrix equation in the single-excitation basis, de-excitations are included due to the squared nature of the matrix.^26,29^

Evaluating the derivative in eq 1 from eq 2 we have

7 $$n_{I}\left(r\right)=n_{0}\left(r\right)+\frac{\delta \omega_{I}}{\delta v_{\mathrm{ext}}\left(r\right)}$$

where the first term, the ground-state density, comes from the derivative of the ground-state energy. We focus here on the excited-state density difference

8 $$\Delta n_{I}\left(r\right)\equiv n_{I}\left(r\right)-n_{0}\left(r\right)=\frac{\delta \omega_{I}}{\delta v_{\mathrm{ext}}\left(r\right)}=\frac{1}{2\omega_{I}}\frac{\delta \omega_{I}^{2}}{\delta v_{\mathrm{ext}}\left(r\right)}$$

obtaining the derivative from eq 3 in the following way. Multiplying by **G**_*I*_^†^ on the left, and then taking the derivative, yields the left-hand side as

9 $$\begin{array}{l} \frac{\delta }{\delta v_{\mathrm{ext}}\left(r\right)}\left(G_{I}^{†}\Omega \left(\omega_{I}\right)G_{I}\right)=\frac{\delta G_{I}^{†}}{\delta v_{\mathrm{ext}}\left(r\right)}\Omega \left(\omega_{I}\right)G_{I}+G_{I}^{†}\Omega \left(\omega_{I}\right)\frac{\delta G_{I}}{\delta v_{\mathrm{ext}}\left(r\right)}+G_{I}^{†}\frac{\delta \Omega \left(\omega_{I}\right)}{\delta v_{\mathrm{ext}}\left(r\right)}G_{I}=\omega_{I}^{2}\frac{\delta }{\delta v_{\mathrm{ext}}\left(r\right)}\left(G_{I}^{†}G_{I}\right)+G_{I}^{†}\frac{\delta \Omega \left(\omega_{I}\right)}{\delta v_{\mathrm{ext}}\left(r\right)}G_{I} \end{array}$$

­(where we have used the Hermitian property of Ω­(ω)) while the right-hand side gives

10 $$\frac{\delta }{\delta v_{\mathrm{ext}}\left(r\right)}\left(\omega_{I}^{2}G_{I}^{†}G_{I}\right)=\omega_{I}^{2}\frac{\delta }{\delta v_{\mathrm{ext}}\left(r\right)}\left(G_{I}^{†}G_{I}\right)+\frac{\delta \omega_{I}^{2}}{\delta v_{\mathrm{ext}}\left(r\right)}G_{I}^{†}G_{I}$$

Equating the two sides gives

11 $$\Delta n_{I}\left(r\right)=\frac{1}{2\omega_{I}}\frac{\delta \omega_{I}^{2}}{\delta v_{\mathrm{ext}}\left(r\right)}=\frac{1}{2\omega_{I}}G_{I}^{†}\frac{\delta \Omega \left(\omega_{I}\right)}{\delta v_{\mathrm{ext}}\left(r\right)}G_{I}/N$$

where $N=G_{I}^{†}G_{I}$ . This expression gives us the exact density difference, once we compute the functional derivative of the TDDFT matrix.

While eq 11 is independent of the choice of normalization N of the response eigenvectors **G**_*I*_ (as it must be from eq 8), we will consider the particular normalization derived in ref 26 which directly relates the eigenvectors to the oscillator strengths via $f_{I}=\frac{2}{3}\omega_{I}\left|\left\langle \Psi_{0}\right|r\right|\Psi_{I}\rangle {|}^{2}=\frac{2}{3}{\left|\sum_{q,q\prime }r_{q}^{†}S_{q,q\prime }^{-1/2}G_{I,q\prime }\right|}^{2}$ ,^27^ where *S* is the diagonal matrix $S_{\mathrm{qq}\prime }=\delta_{\mathrm{qq}\prime }\frac{1}{\nu_{q}}$ . Reference 26 showed that the required normalization condition is

12 $$G_{I}^{†}\left(1̂-\frac{\partial \Omega \left(\omega \right)}{\partial \left(\omega^{2}\right)}{|}_{\omega =\omega_{I}}\right)G_{I}=1$$

Solving this for N and inserting into eq 11 yields

13 $$\Delta n_{I}\left(r\right)=\frac{1}{2\omega_{I}}{\left(1̂+G_{I}^{†}\frac{\partial \Omega \left(\omega \right)}{\partial \left(\omega^{2}\right)}{|}_{\omega =\omega_{I}}G_{I}\right)}^{-1}G_{I}^{†}\frac{\delta \Omega \left(\omega_{I}\right)}{\delta v_{\mathrm{ext}}\left(r\right)}G_{I}$$

We note that the functional derivative $\frac{\delta \Omega \left(\omega_{I}\right)}{\delta v_{\mathrm{ext}}\left(r\right)}$ has two contributions evident from eq 4: one is from the variation of KS orbitals and eigenvalues {ϕ_*i*_(**r**), ϵ_*i*_} that explicitly appear in eq 4 and from the variation of the functional-argument *n*_0_ of the *f*_XC_ kernel under small changes of the external potential, and the other is from the possible frequency-dependence of the xc kernel *f*_XC_(ω) because this is evaluated at the solution point of eq 3, ω_*I*_, which depends on *v*_ext_(**r**), $\frac{\delta \omega_{I}}{\delta v_{\mathrm{ext}}\left(r\right)}$ . Within the commonly used adiabatic approximation, the TDDFT matrix is frequency-independent and so $\frac{\delta f_{\mathrm{XC}}\left(\omega \right)}{\delta \omega }=0$ and $\frac{\partial \Omega }{\partial \left(\omega^{2}\right)}$ in eq 13 is zero. In the general case, frequency dependence of the exact kernel brings nonlinearity to eq 3, resulting therefore in more solutions than the size of the KS single-excitation matrix. This is essential to correctly get states corresponding to states of double-excitation character,^30–34^ which are not accessible with an adiabatic approximation. While the frequency dependence of the *f*_XC_ kernel also affects the density-difference dependence through eq 13, this equation is, however, still linear, as the matrix is evaluated at the solution frequency ω_*I*_, yielding a unique and well-defined density difference in eq 8.

A simplification arises once we separate the two contributions to the functional derivative of the *f*_XC_ kernel that were mentioned above

14 $$\frac{\delta }{\delta v_{\mathrm{ext}}\left(r\right)}f_{\mathrm{HXC}}\left[n_{0}\right]\left(r^{\prime },r^{\prime \prime },\omega_{I}\left[n_{0}\right]\right)={{\frac{\delta f_{\mathrm{HXC}}\left[n_{0}\right]\left(r^{\prime },r^{\prime \prime },\omega \right)}{\delta v_{\mathrm{ext}}\left(r\right)}|}_{\omega =\omega_{I}}+\frac{\partial f_{\mathrm{HXC}}\left(r^{\prime },r^{\prime \prime },\omega \right)}{\partial \omega^{2}}|}_{\omega =\omega_{I}}\frac{\delta \omega_{I}^{2}}{\delta v_{\mathrm{ext}}\left(r\right)}$$

where the second term is only nonzero in the case of nonadiabatic functionals. Transferring this separation to the full Ω­(ω) matrix, and inserting into eq 13, we find

15 $$\Delta n_{I}\left(r\right)=\frac{1}{2\omega_{I}}\frac{\delta \omega_{I}^{2}}{\delta v_{\mathrm{ext}}\left(r\right)}=\frac{1}{2\omega_{I}}G_{I}^{†}\frac{\delta \Omega \left(\omega \right)}{\delta v_{\mathrm{ext}}\left(r\right)}{|}_{\omega =\omega_{I}}G_{I}$$

There are two advantages of using eq 15 over eq 11. First, with the normalization eq 12, the derivative on the right-hand side of the resulting eq 15 no longer includes the variation of the solution point ω_*I*_ under *v*_ext_. Second, eq 15 lends a physical interpretation, in that the factor |*G*_*I,q*_|^2^ multiplying each diagonal term of the derivative matrix can be interpreted as a fraction of the single excitation *q* in the density difference of the true excited state *I*. We will use eq 15 in what follows.

Equations 15 or 11 define the exact excited-state densities once the terms of the derivative matrix $\frac{\delta \Omega_{\mathrm{qq}\prime }\left(\omega \right)}{\delta v_{\mathrm{ext}}\left(r\right)}$ are calculated. When KS states are considered real, one finds

$$\frac{\delta \Omega_{\mathrm{qq}\prime }\left(\omega \right)}{\delta v_{\mathrm{ext}}\left(r\right)}=\int d^{3}x\left\{2\nu_{q}\Delta n_{q}^{\mathrm{KS}}\left(x\right)\delta_{\mathrm{qq}\prime }+2f_{\mathrm{HXC},\mathrm{qq}\prime }\left(\omega \right)\left(\sqrt{\frac{\nu_{q\prime }}{\nu_{q}}}\Delta n_{q}^{\mathrm{KS}}\left(x\right)+\sqrt{\frac{\nu_{q}}{\nu_{q\prime }}}\Delta n_{q\prime }^{\mathrm{KS}}\left(x\right)\right)+4\sqrt{\nu_{q}\nu_{q\prime }}\left(\overset{\infty }{\underset{p\neq i}{\sum }}\frac{1}{\epsilon_{i}-\epsilon_{p}}{f_{\mathrm{HXC}}}_{,\mathrm{pa},\mathrm{jb}}\left(\omega \right)\Phi_{\mathrm{ip}}\left(x\right)+\overset{\infty }{\underset{p\neq a}{\sum }}\frac{1}{\epsilon_{a}-\epsilon_{p}}{f_{\mathrm{HXC}}}_{,\mathrm{ip},\mathrm{jb}}\left(\omega \right)\Phi_{\mathrm{pa}}\left(x\right)+\overset{\infty }{\underset{p\neq j}{\sum }}\frac{1}{\epsilon_{j}-\epsilon_{p}}{f_{\mathrm{HXC}}}_{,\mathrm{ia},\mathrm{pb}}\left(\omega \right)\Phi_{\mathrm{jp}}\left(x\right)+\overset{\infty }{\underset{p\neq b}{\sum }}\frac{1}{\epsilon_{b}-\epsilon_{p}}{f_{\mathrm{HXC}}}_{,\mathrm{ia},\mathrm{jp}}\left(\omega \right)\Phi_{\mathrm{pb}}\left(x\right)\right)\right\}{\left(1̂-f_{\mathrm{HXC}}\chi_{S}\right)}^{-1}\left(x,r\right)+4\sqrt{\nu_{q}\nu_{q\prime }}\int d^{3}x{\mathrm{g̃}}_{\mathrm{XC},\mathrm{qq}\prime }\left(x,\omega \right)\chi \left(x,r\right)$$ 16

Section 1 in the Supporting Information contains the details of the derivation of eq 16. Here we have used the following short-hands. First, we note that when χ, χ_S_, *f*_HXC_ are written without any frequency dependence, this indicates they are to be evaluated at the static limit ω = 0:

$$\chi \left(r,r^{\prime }\right)=\frac{\delta n\left(r\right)}{\delta v_{\mathrm{ext}}\left(r^{\prime }\right)}{|}_{n=n_{0}},\chi_{S}\left(r,r^{\prime }\right)=\frac{\delta n\left(r\right)}{\delta v_{S}\left(r^{\prime }\right)}{|}_{n=n_{0}},\;f_{\mathrm{HXC}}=f_{\mathrm{HXC}}\left[n_{0}\right]\left(r,r^{\prime }\right)=\frac{\delta v_{\mathrm{HXC}}\left[n\right]\left(r\right)}{\delta n\left(r^{\prime }\right)}{|}_{n=n_{0}}$$

In contrast, *f*_HXC_(ω) = *f*_HXC_[*n*_0_]­(**r**, **r**′, ω) is defined as in eq 6. The KS approximation to the single-excitation density difference is denoted Δ*n*_*q*_^KS^(**x**) = ϕ_*a*_^2^(**x**) – ϕ_*i*_^2^(**x**), while Φ_*mn*_(**x**) = ϕ_*m*_(**x**)­ϕ_*n*_(**x**) is defined in the same way as the KS transition density earlier was, except now generalized since ϕ_*m*_, ϕ_*n*_ could both be occupied or unoccupied orbitals. Note that *p* in the sums run over all states, occupied and unoccupied, so this expression probes the xc kernel matrix elements (eq 5) in a much larger range than needed in the calculation of the excitation spectra; the latter involves matrix elements only between single excitations. The real-space matrix $(1̂-f_{\mathrm{HXC}}\chi_{S}{)}^{-1}\left(x,r\right)$ is defined using the static *f*_HXC_ kernel and KS response function, and the product *f*_HXC_χ_S_ indicates the convolution, *f*_HXC_χ_S_(**x**, **r**) = ∫d^3^ *x*′*f*_HXC_(**x**, **x**′)­χ_S_(**x**′, **r**).

Finally, *g̃*_XC,*qq*′_(**x**, ω) = ∫d^3^ *x*′ d^3^ *x*″*g̃*_XC_[*n*_0_]­(**x**′, **x**″, **x**, ω)­Φ_*q*_(**x**′)​Φ_*q*′_(**x**″) is the matrix in KS single-excitation space of

17 $${\mathrm{g̃}}_{\mathrm{XC}}\left[n_{0}\right]\left(x^{\prime },x^{\prime \prime },x,\omega \right)=\frac{\delta f_{\mathrm{XC}}\left[n_{0}^{\prime }\right]\left(x^{\prime },x^{\prime \prime },\omega \right)}{\delta n_{0}^{\prime }\left(x\right)}{|}_{n_{0}^{\prime }=n_{0}}$$

We note that this is a distinct object from the second-order response kernel that appears in quadratic response theory, which is the Fourier transform of the functional derivative $g_{\mathrm{XC}}\left[n_{0}\right]\left(x^{\prime },x^{\prime \prime },x,t-t^{\prime },t-t^{\prime \prime }\right)=\frac{\delta f_{\mathrm{XC}}\left[n_{0}^{\prime }\right]\left(x^{\prime },x^{\prime \prime },t-t^{\prime }\right)}{\delta n_{0}^{\prime }\left(x,t-t^{\prime \prime }\right)}{|}_{n_{0}^{\prime }=n_{0}}$ .

Equations 15–16, once added to the ground-state density, give in principle the exact excited-state density for the state *I*. As such eq 16 must integrate to zero. To see that it does, consider the Taylor expansions of the inverse

$$(1̂-f_{\mathrm{HXC}}\chi_{S}{)}^{-1}=1̂+f_{\mathrm{HXC}}\chi_{S}+f_{\mathrm{HXC}}\chi_{S}\;f_{\mathrm{HXC}}\chi_{S}+\cdot \cdot \cdot$$ 18

and that for the Dyson equation for χ­(**x**, **r**) (see also the Supporting Information). One then observes that the **r**-dependence in all the terms in $\frac{\delta \Omega_{\mathrm{qq}\prime }\left(\omega \right)}{\delta v_{\mathrm{ext}}\left(r\right)}$ involve KS density differences Δ*n*_*q*_^KS^(**r**) or KS transition densities Φ_*mn*_(**r**), and thus integrate to zero.

We note that refs 4,5,35,36 begin instead with finding stationary points of a Lagrangian that expresses the variational property of the excited-state energy, working in the KS single-excitation subspace to directly obtain matrix equations for the so-called *Z*-vector that contains relaxation contributions to a difference density matrix; solving these equations yields excited-state gradients and other properties. Our eqs 15–16 are equivalent but instead yields the densities directly in real-space, and moreover, applies also to the case of nonadiabatic kernels.

### 2.1 Small-Matrix Approximation

While eqs 12 and 15–16 provide the exact prescription for the excited-state density difference, we will make a diagonal approximation to the TDDFT matrix eq 3 to simplify the calculation in our examples. Neglecting the mixing between different single KS excitations is known as the Small-Matrix Approximation (SMA),^29^ and taking the diagonal element of eq 3 yields

19 $$\omega_{I}^{2}\overset{\mathrm{SMA}}{\approx }\nu_{q}^{2}+4\nu_{q}\;{f_{\mathrm{HXC}}}_{\mathrm{qq}}\left(\omega_{I}\right)$$

The utilization of SMA for the excited-state densities has limitations similar to those observed for the excitation frequencies: it performs best when the excited state is dominated by a single KS transition. If the excitation involves a significant contribution from multiple KS states, the densities arising from SMA will be less accurate.

Under SMA, with normalization condition eqs 12, 15 reduces to

20 $$\Delta n_{I}^{\mathrm{SMA}}\left(r\right)=\frac{G_{I}^{2}}{2\omega_{I}}\frac{\delta \Omega_{\mathrm{qq}}\left(\omega \right)}{\delta v_{\mathrm{ext}}\left(r\right)}{|}_{\omega =\omega_{I}}$$

with the eigenvector *G*_*I*_ reducing to the number

21 $$G_{I}=\frac{1}{\sqrt{1̂-\frac{\partial \Omega_{\mathrm{qq}}\left(\omega \right)}{\partial \omega^{2}}{|}_{\omega =\omega_{\mathrm{SMA},I}}}}$$

Inserting the diagonal elements of eq 16 directly into eq 20 leads to the SMA density difference

22 $$\Delta n_{I}^{\mathrm{SMA}}\left(r\right)=\frac{G_{I}^{2}}{\omega_{I}}\int d^{3}x\left\{[\left(\nu_{q}+2{f_{\mathrm{HXC}}}_{\mathrm{qq}}\left(\omega_{I}\right)\right)\Delta n_{I}^{\mathrm{KS}}\left(x\right)+4\sum_{p\neq a}^{\infty }\frac{\nu_{q}}{\epsilon_{a}-\epsilon_{p}}{f_{\mathrm{HXC}}}_{\mathrm{ip},\mathrm{ia}}\left(\omega_{I}\right)\Phi_{\mathrm{pa}}\left(x\right)-4\sum_{p\neq i}^{\infty }\frac{\nu_{q}}{\epsilon_{p}-\epsilon_{i}}{f_{\mathrm{HXC}}}_{\mathrm{pa},\mathrm{ia}}\left(\omega_{I}\right)\Phi_{\mathrm{ip}}\left(x\right)]\times {\left(1̂-f_{\mathrm{HXC}}\chi_{S}\right)}^{-1}\left(x,r\right)+2\nu_{q}{\mathrm{g̃}}_{\mathrm{XC},\mathrm{ia},\mathrm{ia}}\left[n_{0}\right]\left(x,\omega_{I}\right)\chi \left(x,r\right)\}\right\}$$

While this expression is formally derived from a single matrix element, it contains information about all KS states of the system through χ_S_ and the infinite sums over all KS orbitals ϕ_*p*_. We also define a “single-transition limit” (STL) approximation which keeps only KS orbitals *i* and *a* in these terms. STL would be exact in the special case of a two-electron two-level KS system. In this limit, a further simplification ensues: the functions $(1̂-f_{\mathrm{HXC}}\chi_{S}{)}^{-1}$ and χ can be exactly defined by the closed-form expressions

23 $${\left(1̂-f_{\mathrm{HXC}}\chi_{S}\right)}^{-1}\left(x,r\right)=\delta \left(x-r\right)-\frac{4}{\nu_{q}+4f_{\mathrm{HXC},\mathrm{qq}}}f_{\mathrm{HXC},q}\left(x\right)\Phi_{q}\left(r\right)$$

24 $$\chi \left(x,r\right)=\frac{\nu_{q}}{\nu_{q}+4f_{\mathrm{HXC},\mathrm{qq}}}\chi_{S}\left(x,r\right)$$

resulting from a series resummation described in Section 2 of Supporting Information. Under this two-level reduction, one obtains the density difference expression in the following form

25 $$\Delta n_{I}^{\mathrm{STL}}\left(r\right)=\frac{G_{I}^{2}}{\omega_{I}}\left\{\left(\nu_{q}+2f_{\mathrm{HXC},\mathrm{qq}}\left(\omega_{I}\right)\right)\Delta n^{\mathrm{KS}}\left(r\right)+\frac{8}{\nu_{q}+4f_{\mathrm{HXC},\mathrm{qq}}}\left[\left(\nu_{q}+f_{\mathrm{HXC},\mathrm{qq}}\left(\omega_{I}\right)\right)\left(f_{\mathrm{HXC},\mathrm{ii},\mathrm{ia}}\left(\omega_{I}\right)-f_{\mathrm{HXC},\mathrm{aa},\mathrm{ia}}\left(\omega_{I}\right)\right)-\nu_{q}{\mathrm{g̃}}_{\mathrm{XC},\mathrm{qqq}}\left(\omega_{I}\right)\right]\Phi_{q}\left(r\right)\right\}$$

We note that no other approximations are applied in the derivation of eq 25. For an adiabatic approximation to the kernel, this result is formally equivalent to the density-difference matrix representation given by ref 5 in its single-transition limit; see Supporting Information for the details.

#### 2.1.1 Numerical Evaluation of Δ*n*_*I*_^SMA^(**r**)

The calculation of the density difference through eq 16 or its SMA approximation through eq 22 requires addressing several issues, introducing approximations along the way.

First, we will truncate the infinite sums over all KS orbitals (*p*) in eq 22. Since we will be focusing only on the lower excitations, this is not a severe approximation, and convergence can be easily checked.

Second, concerning the *f*_HXC_ matrix elements: As noted earlier, the required matrix elements are not only between the pairs of occupied-unoccupied transitions that are the usual ingredients of linear response calculations. The larger span does not pose a problem for adiabatic *f*_HXC_ approximations: because this is obtained from the second functional derivative of a ground-state energy functional, its form in real-space is known so any matrix element could be computed. However, it is a problem for existing frequency-dependent *f*_HXC_ approximations, such as those relevant to states of double-excitation character, because only the matrix elements involving the KS states that significantly contribute to these states are known.^31,33,34,37^ We note that the TDDFT kernels derived from the Bethe–Salpeter equation (BSE)^38–40^ do provide this larger class of matrix elements, however, their practical use is limited due to computational cost. Unlike most known TDDFT kernels, the frequency-dependent *f*_HXC_ arising from contracting the four-point BSE kernel does not explicitly depend on the electron density, and functional derivatives with respect to the external potential of the resulting matrix elements would be directly computed.

Thus, when we compute the densities of excited states of double-excitation character, we will instead work directly with the excitation energies provided by the dressed frequency-dependent kernel of refs 31,33,34,37 taking their functional derivative directly; we defer a discussion of this to Section 2.2. All other calculations will utilize an adiabatic approximation for both *f*_HXC_(ω) and *g̃*_XC_(ω). This means that the eigenvector squared value *G*_*I*_^2^ in eq 22 reduces to *G*_*I*_^2^ = 1.

The third issue concerns the calculation of the matrix $(1̂-f_{\mathrm{HXC}}\chi_{S}{)}^{-1}$ in eq 22. In nonperiodic systems, the values of $1-f_{\mathrm{HXC}}\chi_{S}$ approach zero at the boundaries of the spatial grid, which serves as a source of instability in many numerical inversion algorithms.^41^ Further, the direct numerical inversion is computationally expensive, so we will evaluate this by assuming *f*_HXC_ is relatively small, and Taylor expand up to first order, in the following way

26 $${\left(1̂-f_{\mathrm{HXC}}\chi_{S}\right)}^{-1}\left(x,r\right)\approx \delta \left(x-r\right)+\int d^{3}x^{\prime }f_{\mathrm{HXC}}\left(x,x^{\prime }\right)\chi_{S}\left(x^{\prime },r\right)$$

27 $$\chi \left(x,r\right)\approx \chi_{S}\left(x,r\right)+\iint d^{3}x^{\prime }d^{3}x^{\prime \prime }\chi_{S}\left(x,x^{\prime }\right)f_{\mathrm{HXC}}\left(x^{\prime },x^{\prime \prime }\right)\chi_{S}\left(x^{\prime \prime },r\right)$$

In most of the cases we have studied, the terms of higher order in *f*_HXC_ of the series eq 26–27 bring a negligibly small correction to the density difference, and we expect this to be generally true for weakly interacting systems. However, as we show in Section 3.2, an inadequate KS approximation of the ground-state quantities of a system can lead to the divergence of the first order Taylor expansion through the KS linear response function χ_S_ if the KS gaps are significantly underestimated.

### 2.2 Densities of States of Double-Excitation Character

With the adiabatic approximation, TDDFT is only able to predict excitations of single-excitation character, composed of linear combinations of one electron promoted from an occupied KS state to a virtual one. The formulation for excited-state densities in refs 4,5 is restricted to this case. For states of double-excitation character, a frequency-dependent kernel was developed^30–34^ that has been shown to perform well on a range of systems,^42^ most recently capturing the curve-crossing between the 1Bu and 2Ag singlet states in trans-butadiene,^34^ for example. The kernel of refs 31,37 operates within a dressed single-pole approximation (DSPA), generalized to a dressed Tamm–Dancoff approximation (DTDA) when more than one single excitation couples to a double, while, in order to obtain meaningful oscillator strengths as well as energies, the kernel of refs 33,34 operates within a dressed small-matrix approximation (DSMA), generalized to the fully dressed TDDFT (DTDDFT). While eq 15 and its SMA eq 22 hold also beyond the adiabatic approximation, they require matrix elements of the kernel that in a larger space than those derived in the approximations, as discussed above. Therefore, we instead take the functional derivative of eq 8 in a different way, directly from the DSPA and DSMA expressions for the excitation energy.

We begin with the DSMA case where^33^

28 $$\omega_{I}^{2}\overset{\mathrm{DSMA}}{\approx }\nu_{q}^{2}+4\nu_{q}f_{\mathrm{HXC},\mathrm{qq}}^{\mathrm{DSMA}}\left(\omega \right)$$

with the frequency-dependent kernel

29 $$f_{\mathrm{HXC},\mathrm{qq}}^{\mathrm{DSMA}}\left(\omega \right)=f_{\mathrm{HXC},\mathrm{qq}}^{A}+\frac{{\left|H_{\mathrm{qD}}\right|}^{2}}{4\nu_{q}}\left(1+\frac{{\left(H_{\mathrm{qq}}+H_{\mathrm{DD}}-2H_{00}\right)}^{2}}{\left[\omega^{2}-\left({\left(H_{\mathrm{DD}}-H_{00}\right)}^{2}+H_{\mathrm{qD}}^{2}\right)\right]}\right)$$

where *f*_HXC,*qq*_^*A*^ is an adiabatic approximation, the matrix elements of the interacting Hamiltonian *H*_*mn*_ are taken in the truncated 3-state Hilbert space that contains ground, singly excited *q* = *i* → *a* and doubly excited *D* = (*j* → *b*, *k* → *c*) states; these states compose the state of interest of double-excitation character, with ν_*bj*_ + ν_*ck*_ = (ϵ_*b*_ – ϵ_*j*_) + (ϵ_*c*_ – ϵ_*k*_) being close to ν_*q*_ = ϵ_*a*_ – ϵ_*i*_, well-separated from other excitations.

Equation 29 is given for the “0” variant of DSMA HXC kernel from the work;^33^ other variants reduce the number of two-electron integrals needed by replacing some of the Hamiltonian matrix elements with either Kohn–Sham or appropriate adiabatic TDDFT values. For example, in the “A” variant, *H*_*qq*_ – *H*_00_ → ω_*q*_^ASMA^ and *H*_*DD*_ – *H*_00_ → ω_*jb*_^ASMA^ + ω_*kc*_^ASMA^, where the frequencies with "ASMA" superscript are given by eq 19 under adiabatic approximation. Here, we have chosen to use the first replacement, but we keep the Hamiltonian matrix element for the double-excitation part. But we note that the variants all give similar results for the system we will study. The two excitation energies given by DSMA eq 28 are therefore

30 $$\omega_{\pm }^{2}=\frac{1}{2}\left\{{\omega^{\mathrm{ASMA}}}^{2}+{\left(H_{\mathrm{DD}}-H_{00}\right)}^{2}+2H_{\mathrm{qD}}^{2}\pm \left(\omega^{\mathrm{ASMA}}+H_{\mathrm{DD}}-H_{00}\right)\sqrt{{\left(\omega^{\mathrm{ASMA}}-\left(H_{\mathrm{DD}}-H_{00}\right)\right)}^{2}+4H_{\mathrm{qD}}^{2}}\right\}$$

Taking the derivative eqs 8 of [Disp-formula eq30] then leads to the DSMA excited-state density

31 $$\Delta n_{\pm }^{\mathrm{DSMA}}\left(r\right)=\frac{1}{2}\left(1\pm \cos\theta \right)\frac{\omega^{\mathrm{ASMA}}}{\Omega_{\pm }}\Delta n^{\mathrm{ASMA}}\left(r\right)+\frac{1}{2}\left(1\mp \cos\theta \right)\frac{H_{\mathrm{DD}}-H_{00}}{\Omega_{\pm }}\frac{\delta \left(H_{\mathrm{DD}}-H_{00}\right)}{\delta v_{\mathrm{ext}}\left(r\right)}+\frac{1}{2\Omega_{\pm }}\left\{\frac{\delta H_{\mathrm{qD}}^{2}}{\delta v_{\mathrm{ext}}\left(r\right)}\pm \sin\theta \frac{\delta }{\delta v_{\mathrm{ext}}\left(r\right)}\left[H_{\mathrm{qD}}\left(\omega^{\mathrm{ASMA}}+\left(H_{\mathrm{DD}}-H_{00}\right)\right)\right]\right\}$$

In the first term above the adiabatic excited-state density difference Δ*n* ^ASMA^ appears, which we obtain from eq 22, with the kernel *f*_HXC_(ω_*I*_) approximated by the chosen adiabatic approximation. This density contributes through the mixing angle θ defined via

32 $$\tan\theta =\frac{2H_{\mathrm{qD}}}{\omega^{\mathrm{ASMA}}-\left(H_{\mathrm{DD}}-H_{00}\right)}$$

that scales linearly with the coupling between states *q* and *D*.

Note that in the first term of eq 31, the adiabatic density difference Δ*n* ^ASMA^(**r**) is scaled by the square of the Casida eigenvector *G*_±_^2^ from eq 21

33 $$G_{\pm }^{2}=\frac{1}{2}\left(1\pm \cos\theta \right)$$

which is a measure of the amount of single-excitation character of the state.^33^ In the limit cos θ = 1, the state indexed by + reduces to a pure double excitation while that indexed by – reduces to a pure single excitation; the opposite is true for cos θ = −1. On the other hand, when cos θ → 0, the states gain an equal contribution of single- and double-excitation character, and become nearly degenerate. Their densities however remain distinct, due to the last term in eq 31.

For the second and third terms of eq 31, which contain nonadiabatic contributions to the density difference, one needs derivatives of the Hamiltonian matrix elements. These can be evaluated from evaluating the Hamiltonian matrix elements via Slater–Condon rules for one-body and two-body integrals and then taking the derivatives (details in Supporting Information)­

34 $$\frac{\delta }{\delta v_{\mathrm{ext}}\left(r\right)}\left\langle \phi_{r}\right|ĥ\left|\phi_{s}\right\rangle =\Phi_{\mathrm{rs}}\left(r\right)+\int d^{3}x\left\{\sum_{p\neq r}^{\infty }\frac{\left\langle \phi_{p}\right|ĥ\left|\phi_{s}\right\rangle }{\epsilon_{r}-\epsilon_{p}}\Phi_{\mathrm{pr}}\left(x\right)+\sum_{p\neq s}^{\infty }\frac{\left\langle \phi_{r}\right|ĥ\left|\phi_{p}\right\rangle }{\epsilon_{s}-\epsilon_{p}}\Phi_{\mathrm{ps}}\left(x\right)\right\}{\left(1̂-f_{\mathrm{HXC}}\chi_{S}\right)}^{-1}\left(x,r\right)$$

35 $$\frac{\delta }{\delta v_{\mathrm{ext}}\left(r\right)}\left(\phi_{r}\phi_{s}|\phi_{m}\phi_{n}\right)=\int d^{3}x\left\{\sum_{p\neq r}^{\infty }\frac{\left(\phi_{p}\phi_{s}|\phi_{m}\phi_{n}\right)}{\epsilon_{r}-\epsilon_{p}}\Phi_{\mathrm{pr}}\left(x\right)+\sum_{p\neq s}^{\infty }\frac{\left(\phi_{r}\phi_{p}|\phi_{m}\phi_{n}\right)}{\epsilon_{s}-\epsilon_{p}}\Phi_{\mathrm{ps}}\left(x\right)+\sum_{p\neq m}^{\infty }\frac{\left(\phi_{r}\phi_{s}|\phi_{p}\phi_{n}\right)}{\epsilon_{m}-\epsilon_{p}}\Phi_{\mathrm{pm}}\left(x\right)+\sum_{p\neq n}^{\infty }\frac{\left(\phi_{r}\phi_{s}|\phi_{m}\phi_{p}\right)}{\epsilon_{n}-\epsilon_{p}}\Phi_{\mathrm{pn}}\left(x\right)\right\}{\left(1̂-f_{\mathrm{HXC}}\chi_{S}\right)}^{-1}\left(x,r\right)$$

where $ĥ=-\frac{1}{2}\nabla^{2}+{\mathrm{v̂}}_{\mathrm{ext}}$ , and the notations are chosen so that ⟨ϕ_*r*_|*ĥ*|ϕ_*s*_⟩ = ∫d^3^ *xϕ*_*r*_(**x**)*h*(**x**)­ϕ_*s*_(**x**) and (ϕ_*r*_ϕ_*s*_|ϕ_*m*_ϕ_*n*_) = ∬d^3^ *x*d^3^ *x*′ϕ_*r*_(**x**)ϕ_*s*_(**x**′) *w*(**x**, **x**′) ϕ_*m*_(**x**′)ϕ_*n*_(**x**′).

While including both excitation and de-excitation components to the TDDFT matrix is necessary to obtain meaningful transition densities,^33^ we will check whether including only the excitation components is adequate for the excited-state density itself, by using the Tamm–Dancoff approximation of DSMA. This is just the dressed single-pole approximation (DSPA) that was derived earlier in ref 31

36 $$\omega_{I}\overset{\mathrm{DSPA}}{\approx }\nu_{q}+2f_{\mathrm{HXC},\mathrm{qq}}^{\mathrm{DSPA}}\left(\omega_{I}\right)$$

where the DSPA kernel is

37 $$f_{\mathrm{HXC},\mathrm{qq}}^{\mathrm{DSPA}}\left(\omega \right)=f_{\mathrm{HXC},\mathrm{qq}}^{A}+\frac{1}{2}\frac{{\left|H_{\mathrm{qD}}\right|}^{2}}{\omega -\left(H_{\mathrm{DD}}-H_{00}\right)}$$

The excited-state density difference following from eq 36 is

38 $$\Delta n_{\pm }^{\mathrm{DSPA}}\left(r\right)=\frac{1}{2}\left(1\pm \cos\theta \right)\Delta n^{\mathrm{ASPA}}\left(r\right)+\frac{1}{2}\left(1\mp \cos\theta \right)\frac{\delta \left(H_{\mathrm{DD}}-H_{00}\right)}{\delta v_{\mathrm{ext}}\left(r\right)}\pm \sin\theta \frac{\delta H_{\mathrm{qD}}}{\delta v_{\mathrm{ext}}\left(r\right)}$$

where Δ*n* ^ASPA^(**r**) is also derived from the derivative of the excited-state energy within the adiabatic SPA (ASPA)

$$\Delta n_{I}^{\mathrm{ASPA}}\left(r\right)=\int d^{3}x\left\{[\Delta n_{I}^{\mathrm{KS}}\left(x\right)+4\overset{\infty }{\underset{p\neq a}{\sum }}\frac{1}{\epsilon_{a}-\epsilon_{p}}f_{\mathrm{HXC}\mathrm{ip},\mathrm{ia}}\Phi_{\mathrm{pa}}\left(x\right)-4\overset{\infty }{\underset{p\neq i}{\sum }}\frac{1}{\epsilon_{p}-\epsilon_{i}}f_{\mathrm{HXC}\mathrm{pa},\mathrm{ia}}\Phi_{\mathrm{ip}}\left(x\right)]\times {\left(1-f_{\mathrm{HXC}}\chi_{S}\right)}^{-1}\left(x,r\right)+2g_{\mathrm{XC}\mathrm{ia},\mathrm{ia}}\left[n_{0}\right]\left(x\right)\chi \left(x,r\right)\right\}$$ 39

It is important to note that, although the factors $\frac{1}{2}\left(1\pm \cos\theta \right)$ appear in the DSPA density difference eq 38, they are not the same as *G*_*I*_^2^ of DSPA, since eq 33 is derived from the normalization eq 12 when the DSMA kernel is used.

## 3 Results and Analysis on Model Systems

We now apply our method to a set of one-dimensional models of two soft-Coulomb interacting electrons with different external potentials. We define the three following external potentials: (i) 1DHe: one-dimensional Helium $v_{\mathrm{ext}}^{1\mathrm{DHe}}\left(x\right)=\frac{-2}{\sqrt{1+x^{2}}}$ to model local excitations, (ii) two double-well models, one in which the ground-state density is largely localized in a soft-Coulomb potential on the left, $v_{\mathrm{ext}}^{\mathrm{gs}‐\mathrm{soft}}\left(x\right)=-\frac{2}{\sqrt{{\left(x+R/2\right)}^{2}+1}}-\frac{1}{\cosh^{2}\left(x-R/2\right)}$ ,^43^ and the other in which the ground-state density is more tightly localized in the left well, $v_{\mathrm{ext}}^{\mathrm{gs}‐\mathrm{loc}}\left(x\right)=-\frac{2}{\sqrt{{\left(x+R/2\right)}^{2}+1}}-\frac{2.9}{\cosh^{2}\left(x+R/2\right)}-\frac{1}{\cosh^{2}\left(x-R/2\right)}$ , where *R* = 7 a.u., and in these wells we will model charge-transfer excitations, and (iii) Harm_γ_: perturbed harmonic $v_{\mathrm{ext}}^{{\mathrm{Harm}}_{\gamma }}\left(x\right)=\frac{1}{2}x^{2}+\gamma \left|x\right|$ for modeling the double excitations, where γ parameter tunes the degree of mixing between a single excitation and double excitation in the second multiplet.

With just two electrons, the Kohn–Sham ground-state has both electrons occupying the lowest-energy orbital *i* = 0, and the KS response function for all three cases can be written as

40 $$\chi_{S}\left(x,x^{\prime }\right)=-4\sum_{a}^{\mathrm{unocc}}\frac{\phi_{0}\left(x\right)\phi_{a}\left(x\right)\phi_{a}\left(x^{\prime }\right)\phi_{0}\left(x^{\prime }\right)}{\epsilon_{a}-\epsilon_{0}}$$

The simplicity of the models enables the computations of the exact KS orbitals, and we can compare the effect of using approximate versus exact KS orbitals in the expressions for the excited-state densities. We will first consider the lowest energy excitations in 1DHe and the two double-wells. These are dominated by single excitations, and so we will consider two adiabatic approximations: exact-exchange (EXX) and local-density approximation (LDA),^44^ which are first-principles approximations at two opposite extremes, the former derived from many-body perturbation theory has nonlocal density-dependence and the latter derived from the uniform electron gas paradigm has completely local density-dependence. We will compare the fully converged SMA densities (eq 22) with those obtained from STL (eq 25). For the Harm_γ_ system, we compare the performance of ASMA using both the EXX and LDA approximations with the DSMA eq 31.

For our calculations of the exact ground and excited-state densities, and the exact, Kohn–Sham, EXX, and LDA orbitals of these one-dimensional systems, we use the Octopus code.^45^ The exact Kohn–Sham potential and orbitals are obtained from first inverting the ground-state Kohn–Sham equation using the exact ground-state density of a two-electron system, and then finding its eigenstates and eigenvalues in an in-house code. The 1DHe model was simulated in a box ranging from −40 to 40 a.u. with a grid spacing of 0.1 a.u. For both double-well models, a box from −50 to 50 a.u. with the same grid spacing was used. Finally, for the system in the harmonic potential, simulations were performed in a box from −20 to 20 a.u. with a grid spacing of 0.05 a.u.

### 3.1 One-Dimensional Helium: Local Excitations

Figure 1 shows the excited-state density differences of the first four local excitations of 1DHe. In this plot, the exact KS orbitals are used in eq 22 and *f*_HXC_ and *g̃*_XC_ are approximated by EXX: $f_{\mathrm{HXC}}\left(x,x^{\prime }\right)=f_{\mathrm{HX}}\left(x,x^{\prime }\right)=\frac{1}{2\sqrt{1+{\left(x-x^{\prime }\right)}^{2}}}$ , and *g̃*_XC_ = *g̃*_X_ = 0. The plots show the fully converged SMA and STL excited-state density differences, as well as the KS one; the inset shows the excited-state density itself, obtained from adding the exact ground-state density to the density difference in each case.

**Figure 1:**
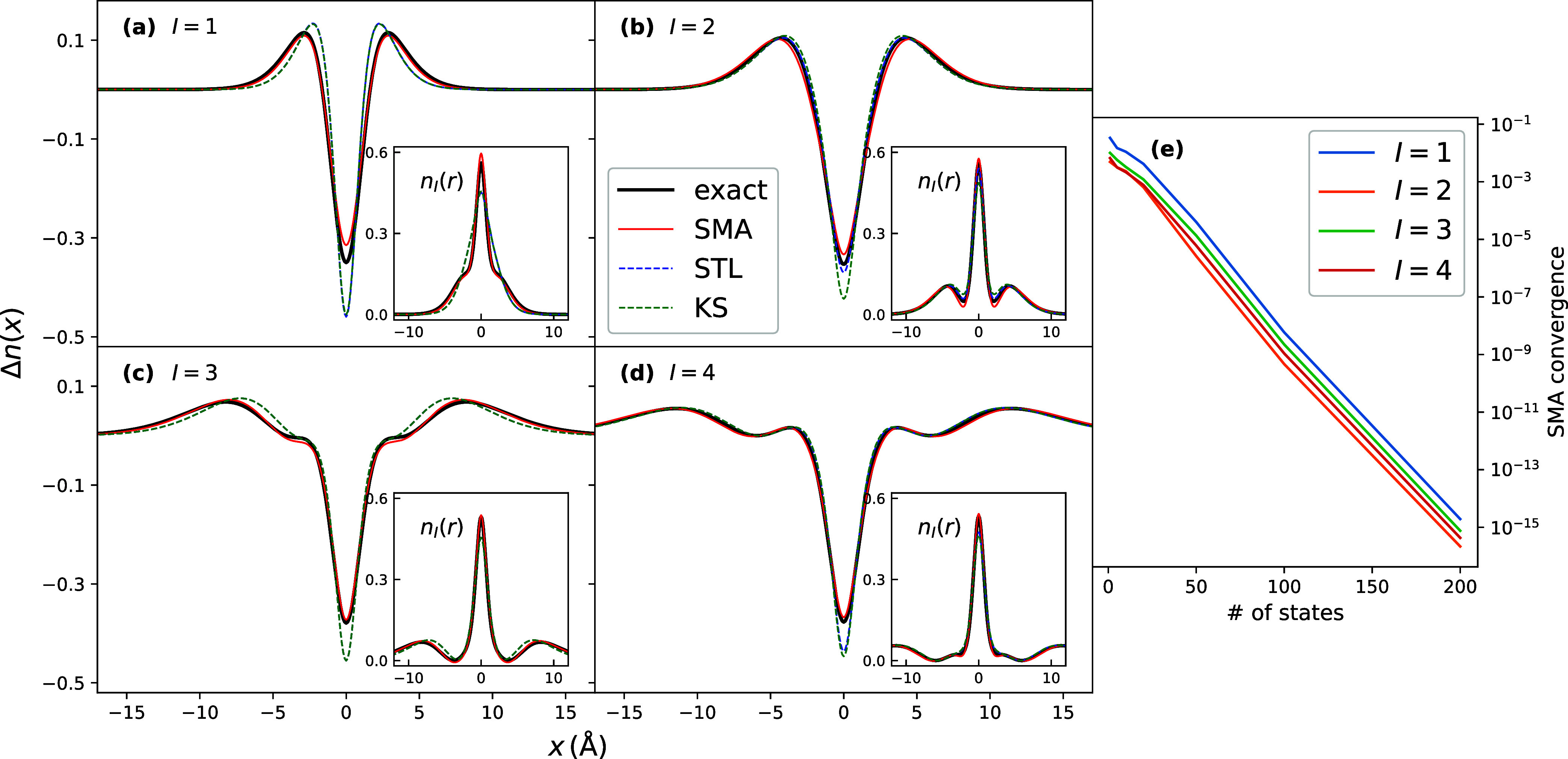
Excited-state density differences for 1D Helium: TDDFT within SMA, STL, compared with exact and KS, for the lowest 4 excitations (panels (a–d)). The insets show the total excited-state density in each case. For the approximations, the exact KS orbitals are used in the formulas for the densities, and *f*_HX_ is used for the kernel. Panel (e) shows the convergence for the lowest 3 excited densities with respect to the number of orbitals included in the sums over *p*.

We see that SMA gives a noticeable correction to the KS excited-state densities, particularly for the lowest excitation, and that the SMA, STL, and KS densities all become more accurate for the higher excitations, as expected. In particular, for the lowest excitation, SMA captures the sharper peak and the shoulders around the location of the He nucleus, which are missed by the KS density in the lowest excitation, as seen in the inset. For this lowest excitation, the effect of the higher KS orbitals is important in the construction of χ_S_ going into the formula, since the STL density is almost indistinguishable from the KS density. For the higher excitations shown, STL brings a small correction to the KS density (in fact, almost none for the third excited state).

Panel (e) shows the convergence of the SMA density difference with the number *K* of orbitals included in the sums over *p* in eq 22, defined as σ_*K*_ = ∫d*x* |Δ*n*_*I*_^*K*^(*x*) – Δ*n*_*I*_^500^(*x*)|^2^, where *N* is the number of grid points. We see that, for these states, even with just one orbital, this spatially averaged error is relatively small, within 0.03 for the lowest excited state, and 0.01 for the second-fourth, and this error decreases rapidly with the number of orbitals, dropping to well within 0.00001 by 50 orbitals.

In Figure 2, we compare the SMA densities predicted with the EXX and LDA approximations for the orbitals and the *f*_HXC_ kernel; in the cases when *f*_HXC_^LDA^ is used, we also use the LDA approximation for *g̃*_XC_ = *g̃*_XC_^LDA^. We find that EXX performs notably better than LDA, particularly for the choice of the KS orbitals. Using *f*_HXC_^LDA^ with EXX orbitals tends to slightly under- and overestimate the density difference near the position of He nucleus, but the effect of the kernel is less than the effect of the choice of the KS orbital approximation. Though SMA with LDA orbitals seems to estimate the density difference only slightly worse for the lowest excitation, for higher ones, it fails to capture the density difference accurately away from the nucleus, as might be expected since the too-rapid decay of the LDA KS potential causes significant errors in the KS orbitals away from the core.

**Figure 2:**
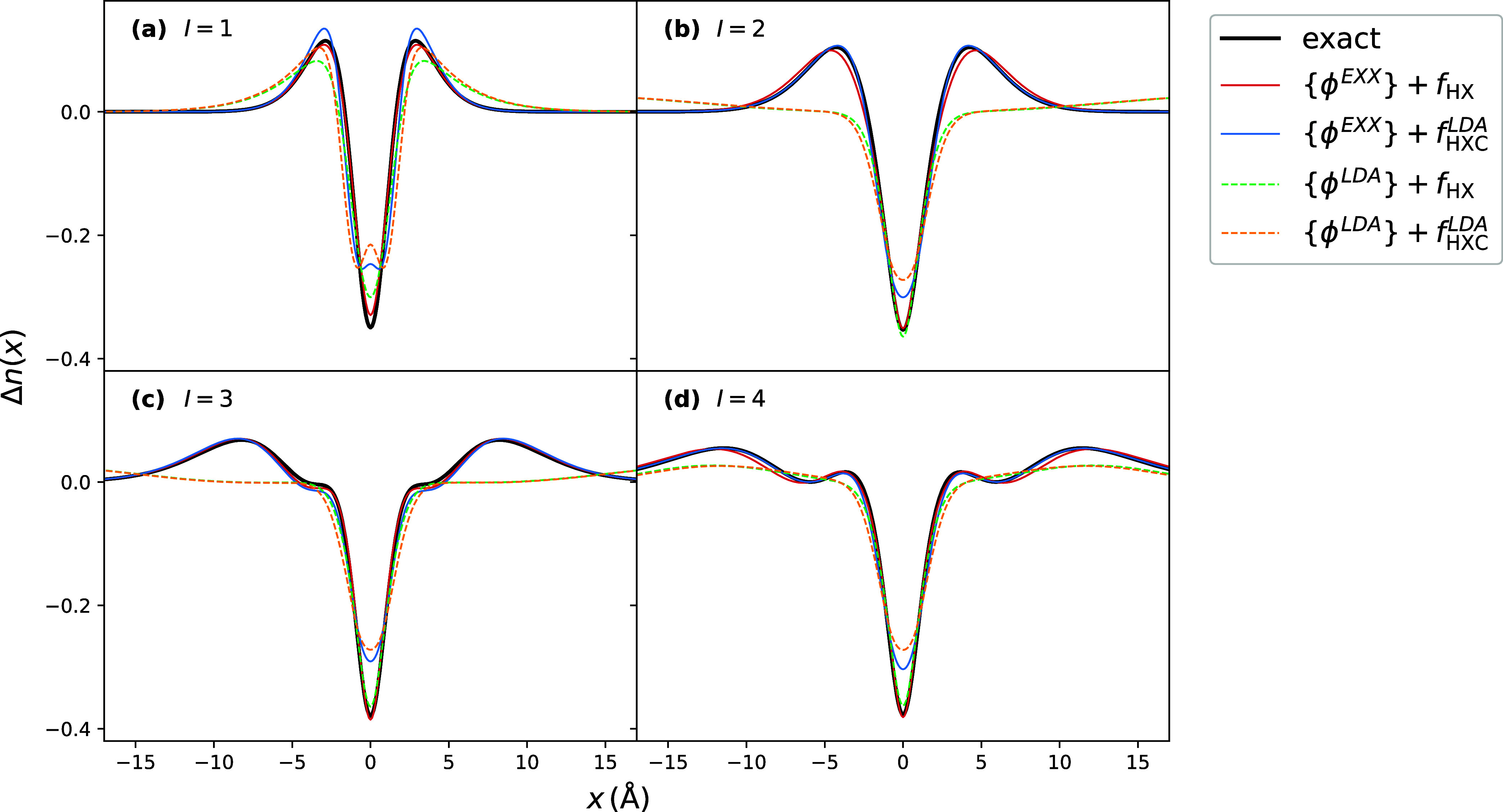
Excited-state density differences for 1D Helium: comparison of approximations for KS orbitals and *f*_HXC_ within SMA. Panels (a–d) correspond to the lowest 4 excitations.

### 3.2 Double-Well Potentials: Charge-Transfer Excitations

We next turn to the lowest charge-transfer excitations in the double-well potential systems. The parameters chosen are such that the ground-state density is localized in the left well.

We begin with the double-well where the ground-state has both electrons in the soft-Coulomb potential on the left (*v*_ext_^gs–soft^), that had been studied in ref 43 The lowest excitation has a charge-transfer nature. In Figure 3(a), we observe that, when exact KS orbitals are used to evaluate the sums in the expressions, the KS density is already a good approximation with only a small error to the density of the charge-transfer excitation, that STL gives practically no correction, while the SMA gives a larger correction, giving a density very close to the exact. The results in this figure used EXX for the kernel.

**Figure 3:**
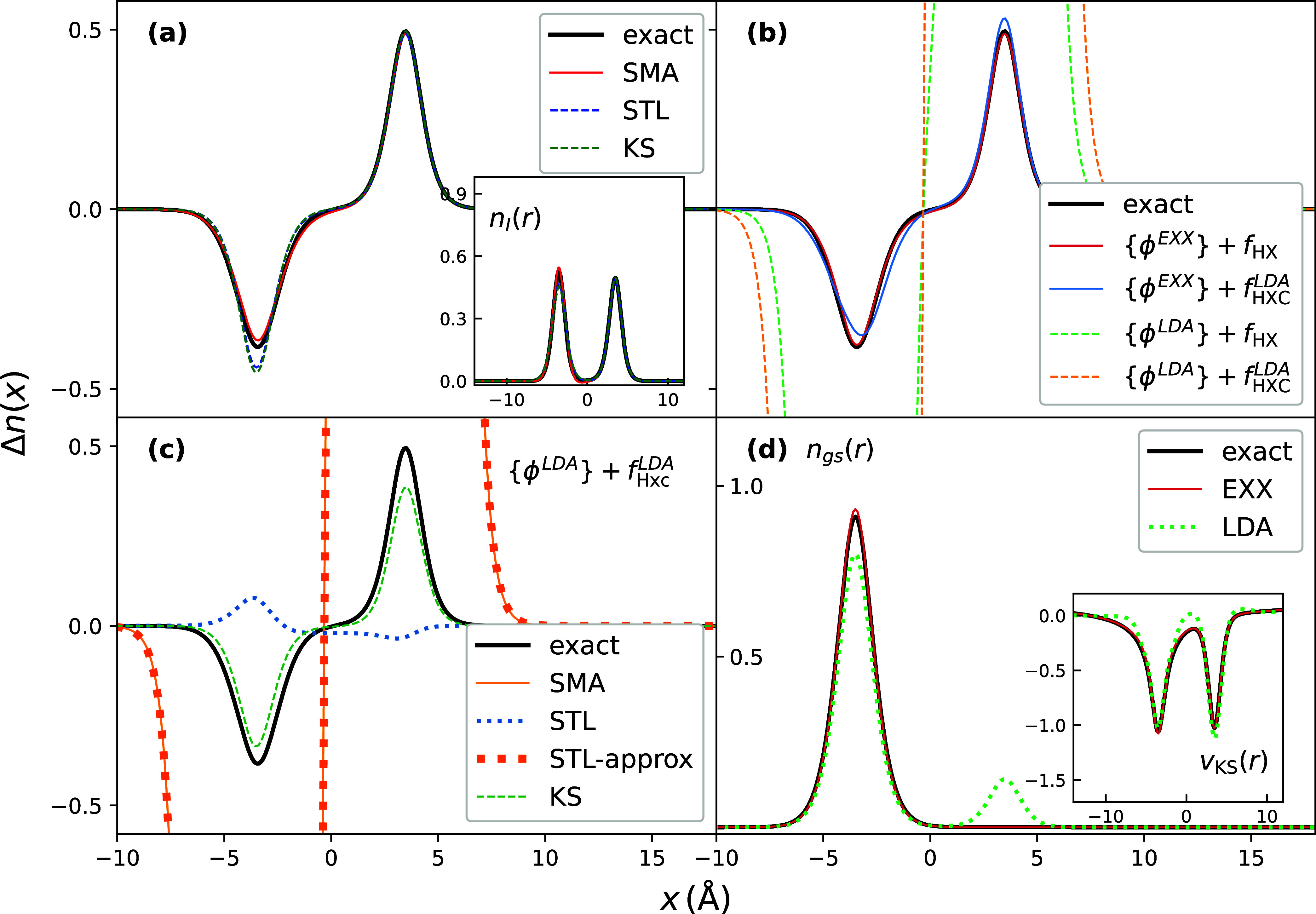
Excited-state density differences for the CT excitation in the double-well model *v*_ext_^gs–soft^.^43^ Panel (a) shows SMA, STL compared to the exact and KS excited-state density difference. Exact KS orbitals and *f*_HX_ were used in approximations. The inset shows the excited-state density of CT state. Panel (b) shows the performance of SMA with KS orbitals and *f*_HXC_ approximated by LDA and EXX functionals. Panel (c) illustrates the issues of SMA and STL: “STL-approx” denotes the excited-state density difference when the first order expansion of $(1̂-f_{\mathrm{HXC}}\chi_{S}{)}^{-1}$ was used as an approximation, while STL with no such approximation shows no divergences. Panel (d) shows the exact, EXX ground-state densities, along with and LDA ground state which has an incorrect delocalization to the right-hand well, and the corresponding KS potentials in the inset.

In Figure 3(b), we compare the performance of approximations for the KS orbitals and the kernel. An immediate observation is that the use of LDA orbitals leads to huge errors with any choice of kernel approximation. Using EXX orbitals gives good results similar to using exact KS orbitals, when either the EXX or LDA kernels are used, with the former kernel being slightly more accurate. In contrast, the use of LDA orbitals leads to completely wrong density differences for the charge-transfer excitation, even yielding negative density in some regions of space, and divergences at the two nuclei.

Why does this problem emerge? LDA underestimates the gap between the occupied and lowest unoccupied KS levels: while the exact LUMO–HOMO gap is ν_exact_ = 0.112 a.u., the LDA gap is far smaller, at ν_LDA_ = 0.005 a.u. This means that for *all* excitations, the terms in eq 22 have much too small denominators, which in itself would lead to divergences unless canceled out by a similar term in the numerator. For this lowest charge-transfer excitation, this cancellation does in fact occur, but the divergence remains because the gap appears also in the denominator of χ_S_. As can be seen in Figure 3(b), this yields a vastly overestimated density in the right well, with an integrated value to the right far larger than the value of 1 that is expected for the charge-transfer state. The density difference is equally too negative in the left well, such that the total difference-density is zero (see eq 18 and discussion there), and the total density unphysically goes negative in the left region. When we approximate $(1̂-f_{\mathrm{HXC}}\chi_{S}{)}^{-1}$ by its first-order term in the Taylor expansion in *f*_HXC_χ_S_, the severely underestimated denominator leads to the divergence. Thus, the problem lies not with the SMA itself, but rather with our approximation of $(1̂-f_{\mathrm{HXC}}\chi_{S}{)}^{-1}$ that appears in it.

Instead, as discussed earlier, the STL approach resums the Taylor series (eq 23) and so treats this inverse exactly, although within the STL approximation. In Figure 3(c), we show that the STL density difference with LDA indeed does not display any divergence, while approximating the inverse function $(1̂-f_{\mathrm{HXC}}\chi_{S}{)}^{-1}$ by its first-order term in *f*_HXC_ in the same way as done in SMA does diverge. However, with LDA, the STL charge-transfer density difference still gives substantial error compared to exact one, and this error stems from the erroneous LDA ground-state shown in Figure 3(d). The LDA suffers from a delocalization error, and has an unphysical fractional charge in each well instead of asymptotically having a two-electron density fully on the left. The ground-state fractional-charge error comes hand in hand with the vanishing LDA KS HOMO–LUMO gap, and this fractional-charge error is transferred to the KS charge-transfer excitation too. This is evident in Figure 3(c) where integrating the KS curve on either side of zero falls short of 1. The wrong behavior of the KS orbitals leads to an erroneous STL density difference, even with the wrong sign.

To emphasize the importance of an accurate KS ground-state, we perform the same calculations on the double-well model with the potential *v*_ext_^gs–loc^ from ref 46 where the two electrons in the ground-state are more tightly localized in the left well, so that LDA has a better hope to capture this qualitatively correctly. The second lowest excitation of this system is the charge transfer, the density of which is shown in Figure 4.

**Figure 4:**
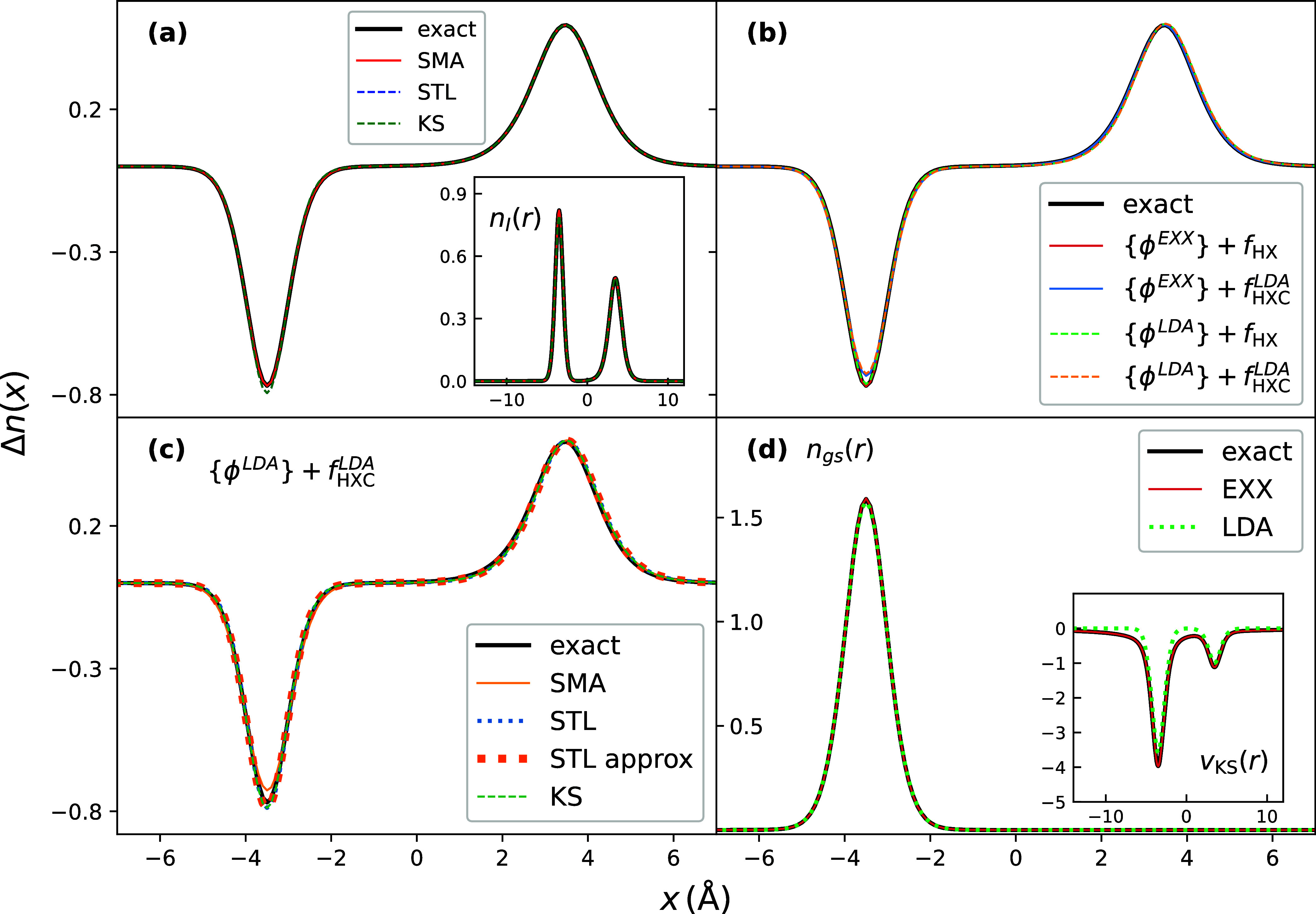
Excited-state density differences for the CT excitation in the double-well model *v*_ext_^gs–loc^.^46^ Panel (a) shows SMA, STL compared to the exact and KS excited-state density difference, using the exact KS orbitals and *f*_HX_. The inset shows the excited-state density of CT state. Panel (b) shows the performance of SMA with KS orbitals and *f*_HXC_ approximated by LDA and EXX functionals as indicated in the legend. Panel (c) illustrates the performance of STL compared to SMA and exact excited-stated density difference. Panel (d) shows the exact, EXX and LDA ground-state densities, and corresponding KS potentials in the inset.

The charge-transfer excited-state density differences obtained with exact KS orbitals and *f*_HX_ (Figure 4(a)), as well as with EXX and LDA approximations (Figure 4(b)), are all in good agreement with the exact. We highlight specifically the result given by LDA approximation, since unlike in the previous result, we not only do not encounter the divergences of the density difference, but it even agrees closely with the exact result. The KS excitation frequency is not qualitatively different from the exact, as it was in the previous case: the exact HOMO–LUMO gap is ν_exact_ = 2.235 a.u., while LDA one is ν_LDA_ = 1.974 a.u. We emphasize that getting reasonable KS quantities is of the most importance for the good performance of the excited-state densities. Regarding the approximate evaluations of these densities, we observe that while both SMA and STL accurately capture the charge transfer in this case, STL performs slightly better. This can be explained by inspecting the behavior of LDA KS potential in the inset of Figure 4(d): the lowest KS orbitals tend to be accurate (so the ground state is accurate), but the excited KS orbitals entering the sums of eqs 22 are not.

### 3.3 Harmonic Potentials: Double Excitations

Turning now to our third model, Harm_γ_, our final example is that of excited-state densities of states of double-excitation character. In this system the lowest excitation is a single excitation, and we expect adiabatic SMA to work particularly well especially since it is well-separated from other excitations. The second and third excitations are mixtures of KS single and double excitations; at γ = 0 the mixture is close to 50:50, so that the first and the second terms of eqs 31 and 8 contribute equally to the density difference of both states while as γ increases to one, the KS near-degeneracy is increasingly lifted, with the lower state of the pair acquiring a predominantly single-excitation character, and the upper state a predominantly double.

Figure 5 shows the excited-state densities of the lowest three excitations, predicted from adiabatic SMA, SPA, frequency-dependent DSMA and DSPA, and compared with the exact. The results in Figure 5 utilize the exact KS orbitals and exact KS orbital energies in the construction of eqs 22, 31 and 38, together with the EXX kernel for the adiabatic part. As expected, the approximations coincide closely with the exact for the lowest excitation, but differ for the second and third due to their double-excitation character. Both DSPA and DSMA do an excellent job, while the adiabatic SMA and SPA qualitatively fail for both states at γ = 0. We note the adiabatic approximation predicts only one state, so the predicted density for both the actual second and third states is the same with the adiabatic SMA/SPA. For the third state at γ = 1 the adiabatic approximations also fail, but they provide a closer approximation for the second state, with a small error at the origin, consistent with the fact that it is dominated by the single excitation. We observe that for DSPA and DSMA, the second excitation performs worse compared to ASPA/ASMA, and we will address that shortly when discussing the performance of LDA kernel utilized with the exact KS orbitals in the same setting.

**Figure 5:**
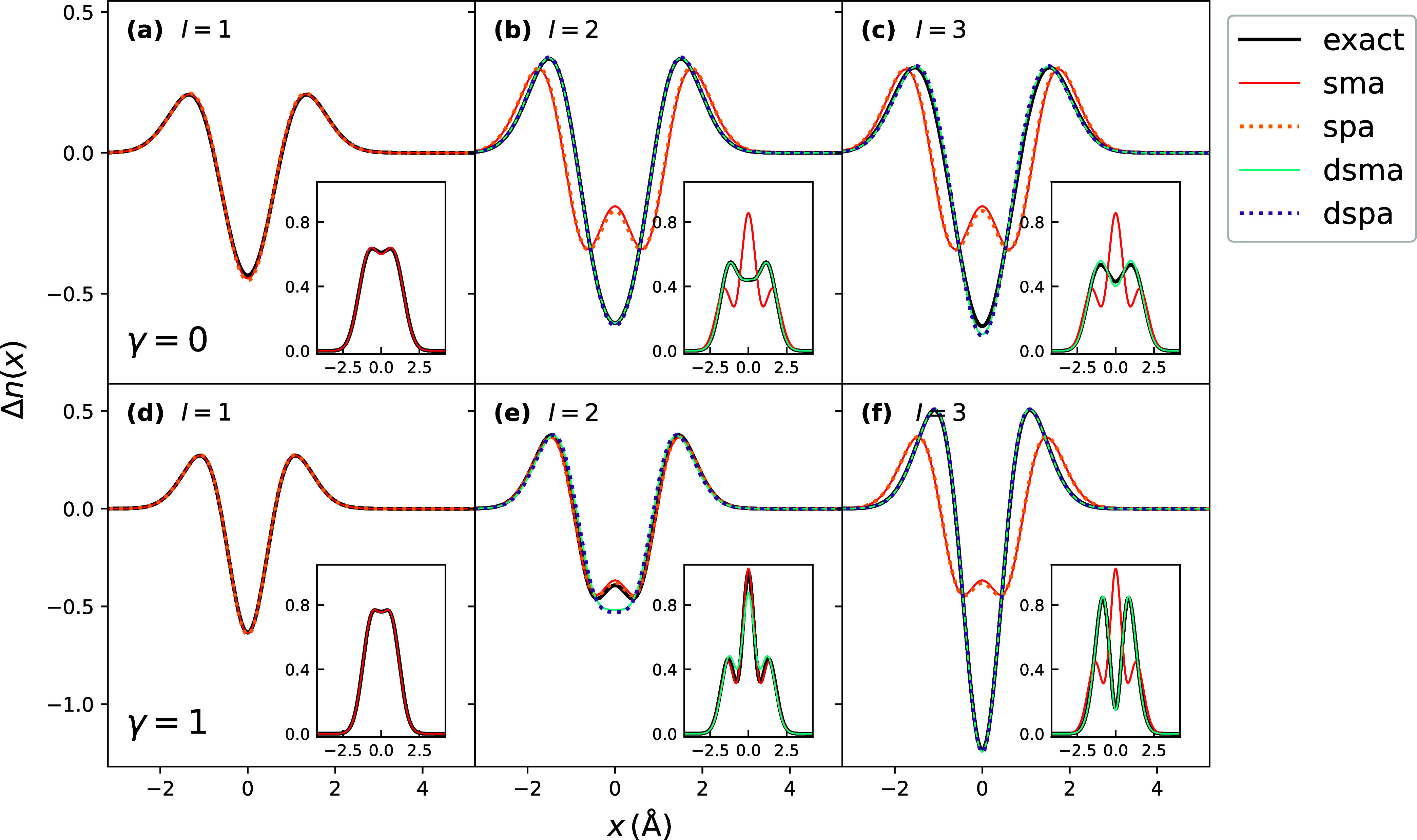
Excited-state densities of the Harm_γ_ model with the set of exact orbitals and EXX kernel. (a) First excitation with γ = 0, showing close agreement of exact, adiabatic SMA and SPA densities. (b, c) Second and third excitations showing close agreement of DSMA and DSPA with exact, and larger errors of adiabatic SMA and SPA, due to the missing double-excitation character of the latter (see text). (d) Lowest excitation with γ = 1, again showing close agreement of exact, adiabatic SMA and SPA densities. (e, f) Second and third excitations with γ = 1 where ASMA outperforms DSMA and DSPA for the second excitation; however, DSMA and DSPA still are in close agreement with the exact result.

In Figure 6, we apply the LDA kernel for *f*_HXC_^*A*^ of eqs 22, 31 and 38, and immediately observe an increased difference between ASPA and ASMA densities, as well as between DPSA and DSMA ones. Upon inspecting the different terms, we find that most of the difference between the ASPA and ASMA appears to be due to the *g̃*_XC_^LDA^ term and this is due to a factor of 2 difference in the χ_S_: in the SPA eq 39, for consistency, we use the response function in the Tamm–Dancoff approximation, which in the static limit is different from the response function eq 40 used in eq 22 by the factor 1/2, that is

41 $$\chi_{S}^{\mathrm{TDA}}\left(r,r^{\prime },\omega =0\right)=-2\sum_{a}^{\mathrm{unocc}}\frac{\phi_{0}\left(x\right)\phi_{a}\left(x\right)\phi_{a}\left(x^{\prime }\right)\phi_{0}\left(x^{\prime }\right)}{\epsilon_{a}-\epsilon_{0}}$$

Note that in the previous figure, where *f*_HX_ was utilized, *g̃*_XC_ = 0, so this difference did not arise. Differences in ASMA and ASPA densities contribute to DSMA and DSPA density differences.

**Figure 6:**
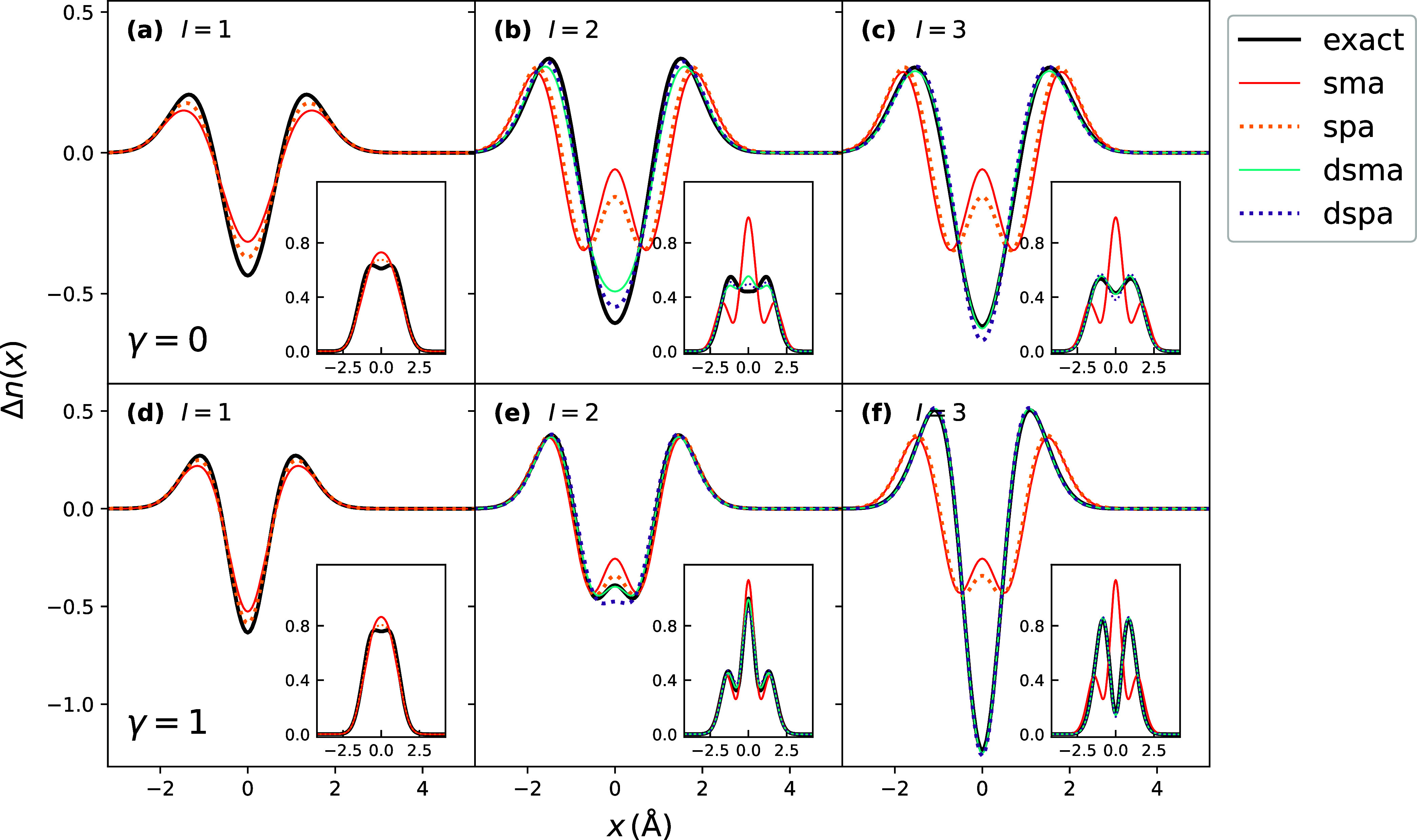
Excited-state densities of the Harm_γ_ model with the set of exact orbitals and LDA kernel. (a) First excitation with γ = 0, where ASPA outperforms ASMA but still exhibiting error at the origin. (b, c) Second and third excitations showing closer agreement of DSPA with exact over DSMA in the second excitation, and vice versa in the third excitation, with qualitatively larger errors of adiabatic SMA and SPA, due to their missing double-excitation character. (d) Lowest excitation with γ = 1, again showing closer agreement of ASPA density with exact over ASMA. (e, f) Second and third excitations with γ = 1 where DSMA outperforms both adiabatic approximations and DSPA in the second excitation, while in the third, both DSPA and DSMA are in close agreement with the exact result.

Interestingly, unlike with the EXX kernel, DSMA with the LDA kernel performs a little worse than DSPA for the second excitation at the limit γ = 0 of Harm_γ_ model, overestimating the density at the origin and underestimating it in the vicinity, while in the limit of γ = 1, it performs best compared to all other approximations. In the latter case, ASPA density outperforms the ASMA one; however, this does not translate to an advantage of DSPA over DSMA. For the third excitation, even though DSPA exhibits an error at the origin, both dressed approximations perform well compared to the exact density.

In Figure 7, we show the DSMA doubly excited-state density differences obtained with the sets of EXX and LDA orbitals used in eq 31 together with EXX and LDA for *f*_HXC_^*A*^. Since both provide accurate approximations for the most relevant KS orbitals, most of the differences in the resulting densities are mostly due to the *f*_HXC_^*A*^ kernel. Therefore, the results obtained do not differ much compared to those shown in Figure 5 for the densities calculated with EXX kernel, and to Figure 6 for those calculated with LDA kernel, both on top of the exact KS orbitals and energies.

**Figure 7:**
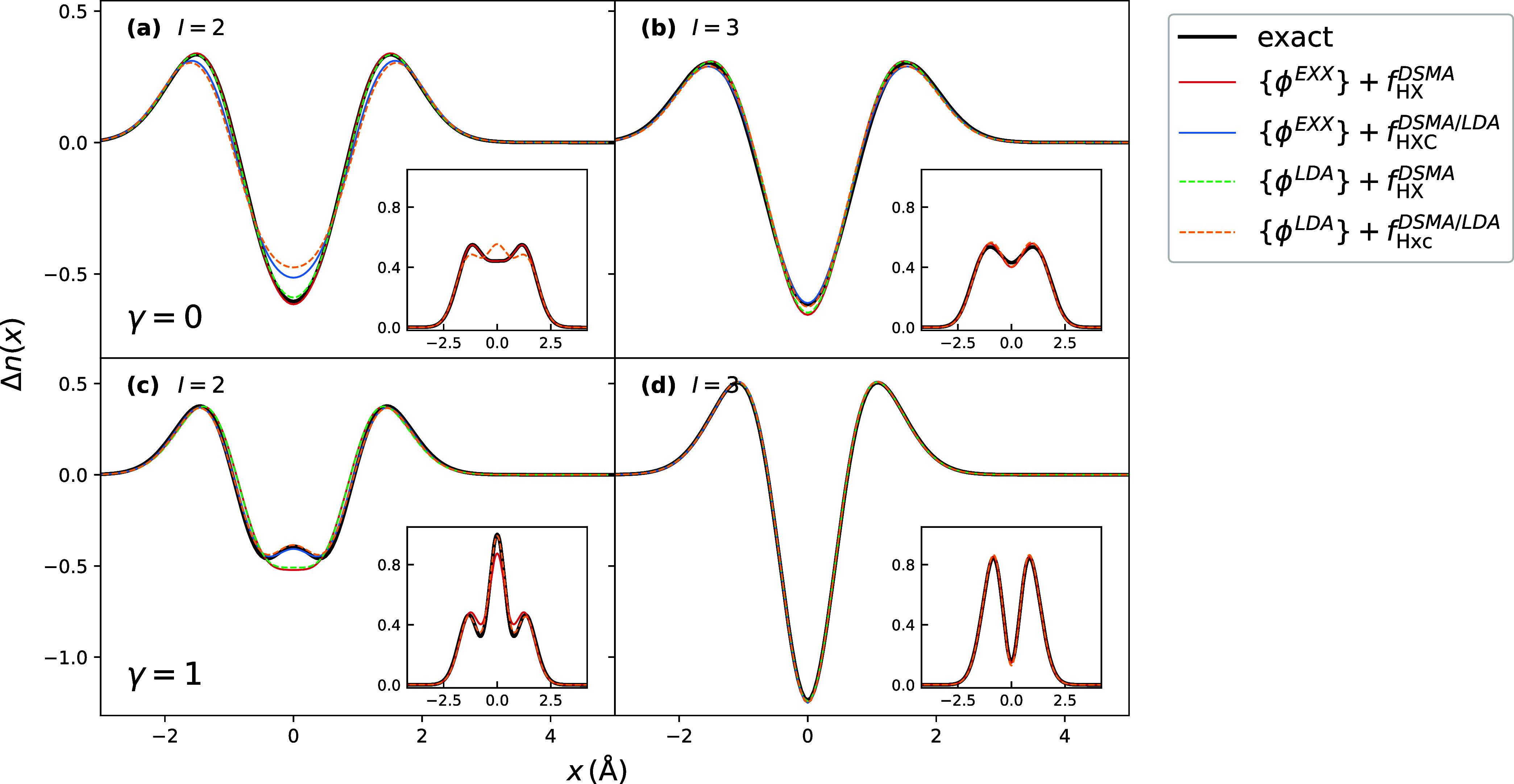
Excited-state density differences for the second and the third excitations of Harm_γ_ model: comparison of approximations for KS orbitals and *f*_HXC_ within DSMA. Panel (a) shows the better agreement of the exact and DSMA densities with EXX kernel over the DSMA with LDA kernel in the second excitation for γ = 0, while Panel (c), on the contrary, shows better performance of LDA kernel in the second excitation for γ = 1. Panels (b, d) show close agreement of DSMA with both *f*_HXC_ in both limits of γ.

Table 1 shows the energies and approximate single-excitation character *G*_*I*_^2^. The (slightly) larger error in the DSMA predictions of the second excited-state density when *f*_HXC_^LDA^ is used for the ingredients in the DSMA in Figures 6(b) and 7(a) is consistent with its larger errors in the excitation energy for the lower of the two states of double-excitation character in the γ = 0 case. Yet, the accuracy of the excitation frequency under *f*_HX_ does not guarantee a better prediction of the corresponding density for γ = 1, as seen in Figures 5(e) and 7(c).

**Table 1:** Comparison of Adiabatic ASMA, DSMA, and DSPA Frequencies and DSMA Single-Excitation Character *G*_*I*_^2^ Using Different Approximations for the Orbitals and Kernels in Equation 31

γ	approximation	ω^adia^	ω_2_^DSPA^	ω_2_^DSMA^	ω_3_^DSPA^	ω_3_^DSMA^	*G*_2_^2^	*G*_3_^2^
γ = 0	exact	–	1.73	1.73	2.00	2.00	0.56	0.44
	{ϕ^exact^} + *f*_HX_	1.86	1.72	1.72	2.01	2.01	0.52	0.48
	{ϕ^exact^} + *f*_HXC_^LDA^	1.83	1.70	1.70	1.99	1.99	0.56	0.44
	{ϕ^EXX^} + *f*_HX_	1.87	1.72	1.72	2.01	2.01	0.50	0.50
	{ϕ^LDA^} + *f*_HXC_^LDA^	1.83	1.70	1.70	1.99	1.99	0.57	0.43
	{ϕ^EXX^} + *f*_HXC_^LDA^	1.84	1.71	1.71	2.00	2.00	0.54	0.46
	{ϕ^LDA^} + *f*_HX_	1.85	1.71	1.72	2.01	2.01	0.52	0.48
γ = 1	Exact	–	2.60	2.60	2.98	2.98	0.88	0.12
	{ϕ^exact^} + *f*_HX_	2.66	2.61	2.61	2.99	2.99	0.85	0.15
	{ϕ^exact^} + *f*_HXC_^LDA^	2.63	2.57	2.57	2.98	2.98	0.88	0.12
	{ϕ^EXX^} + *f*_HX_	2.67	2.62	2.61	2.99	2.99	0.85	0.15
	{ϕ^LDA^} + *f*_HXC_^LDA^	2.63	2.58	2.58	2.98	2.98	0.87	0.13
	{ϕ^EXX^} + *f*_HXC_^LDA^	2.63	2.58	2.58	2.98	2.98	0.87	0.13
	{ϕ^LDA^} + *f*_HX_	2.66	2.61	2.61	2.99	2.99	0.85	0.15

Overall, we have shown that the frequency-dependent kernels of ref 31,33 which were shown earlier to produce good excitation energies for states of double-excitation character, and that of ref 33 also good oscillator strengths, both also yield excellent approximations to excited-state densities. We note that the behavior of the densities under EXX and LDA kernels used for adiabatic part in DSMA is not always consistent with their relative performance for the energies of these states given by the same approach. To understand the underlying reasons of such behavior, further exploration is required. Still, the differences are very small compared to the improvement from the adiabatic description which entirely misses one state.

## 4 Conclusions

In this work we developed a general formalism for obtaining the real-space excited-state densities from linear response TDDFT using time-independent perturbation theory. While the approach is equivalent to earlier work^4,5^ presented using the stationary property of excitation energies and the variational principle, our work here goes beyond the restriction to the adiabatic approximation assumed there. We observe that even with two contrasting approximations (LDA and EXX), we obtain accurate results for the lowest local and charge-transfer excitations between closed-shell fragments. Within the small-matrix approximation, which in its essence retains a single KS transition in its calculation of the energy, the excited-state density still incorporates corrections from all possible KS transitions, leading to a significant improvement over the KS approximation. Our results show the importance of an accurate KS potential in the calculation of excited-state density, which is better provided by adiabatic EXX than the LDA. For the future calculations of excited-state densities in real systems, our results are consistent with previous works^16–22^ that suggest the use of hybrid functionals with large fraction of exact exchange as a more reliable choice.

The practical use of the general formalism applies to the adiabatic approximation and to proposed approximations that are available for frequency-dependent *f*_HXC_ kernels. While a general-purpose frequency-dependent kernel does not exist, the formulas here hold for existing frequency-dependent kernels developed for particular situations. We showed that for doubly excited states, the DSPA and DSMA-based formulations, both previously shown to be good for excitation energies and DSMA for oscillator strengths, provide accurate double-excitation densities, which is not achievable with the adiabatic approximation. The general idea of obtaining excited-state densities from derivatives of energies with respect to the external potential could also be applied to other methods, e.g., pp-RPA,^47,48^ BSE.^49,50^

Benchmarking on a wider set of real molecules is required to further probe the efficiency and accuracy of the linear response formalism developed for real-space excited-state densities for states of both single- and double-excitation character, as has been explored for dipole moments restricted to the adiabatic approximation (single excitations) in earlier works.^16–23^ Beyond static properties, the formalism can be used in the response-reformulated TDDFT for nonequilibrium dynamics,^51^ a key ingredient of which are the densities of the states involved in the dynamics. In this context, fully nonequilibrium dynamics have a propensity to occupy states of double-excitation character during the evolution, whose densities can be obtained from our formulation.

## Supplementary Material



## Notes

Additional details on the derivation of equations throughout the text (PDF)
