# Supplementary material for: Excited-State Densities from Time-Dependent Density Functional Response Theory
Source: J Chem Theory Comput. 2025 Oct 10;21(20):10437–51. doi: 10.1021/acs.jctc.5c00909 (PMC12573763; doi:10.1021/acs.jctc.5c00909)
Supplement: Supplementary file 1 [file ct5c00909_si_001.pdf]

Supporting Information: Excited State Densities from Time-Dependent Density Functional Response Theory

Anna A. Baranova* and Neepa T. Maitra

Department of Physics, Rutgers University, Newark 07102, New Jersey USA

1 Computation of the Derivative Matrix $\frac{\delta\Omega_{qq'}(\omega)}{\delta v_{\rm ext}(\mathbf{r})}$

Excited-state densities are obtained from the functional derivative of the TDDFT linear response matrix

$$\Omega_{qq'}(\omega) = \nu_q^2\delta_{qq'} + 4\sqrt{\nu_q\nu_{q'}}f_{\rm HXC,qq'}(\omega) \tag{1}$$

with respect to external potential $v_{\rm ext}$, evaluated at the TDDFT frequency of the state of interest. We have restricted our analysis to singlet states here and in the main text. The dependence arises from the ground-state density-dependence of $\Omega(\omega) \equiv \Omega[n_0](\omega)$, and the derivative has three terms:

$$\frac{\delta\Omega_{qq'}(\omega)}{\delta v_{\rm ext}(\mathbf{r})} = \frac{\delta\nu_q^2}{\delta v_{\rm ext}(\mathbf{r})}\delta_{qq'} + 4f_{\rm HXC,qq'}(\omega)\frac{\delta}{\delta v_{\rm ext}(\mathbf{r})}\sqrt{\nu_q\nu_{q'}} + 4\sqrt{\nu_q\nu_{q'}}\frac{\delta f_{\rm HXC,qq'}(\omega)}{\delta v_{\rm ext}(\mathbf{r})}\,. \tag{2}$$

To evaluate these, we make use of the functional chain-rule: for an arbitrary functional of the ground-state density $F[n_0]$,

$$\frac{\delta F[n_0]}{\delta v_{\rm ext}(\mathbf{r})} = \iint d^3x d^3x' \frac{\delta F[n_0]}{\delta v_{\rm S}(\mathbf{x})} \frac{\delta v_{\rm S}(\mathbf{x})}{\delta n(\mathbf{x}')}\bigg|_{n=n_0} \frac{\delta n(\mathbf{x}')}{\delta v_{\rm ext}(\mathbf{r})}\bigg|_{v_{\rm ext}=v_{\rm ext}[n_0]} = \iint d^3x d^3x' \frac{\delta F[n_0]}{\delta v_{\rm S}(\mathbf{x})} \chi_{\rm S}^{-1}(\mathbf{x},\mathbf{x}')\,\chi(\mathbf{x}',\mathbf{r}), \tag{3}$$

where χ and $\chi_{\rm S}$ are the static interacting and Kohn-Sham (KS) response functions respectively.

Let us first consider the product of the inverse KS and interacting response functions inside the integral. The non-interacting KS linear response has the following explicit representation in terms of the KS orbitals and energies:

$$\chi_{\rm S}(\mathbf{x},\mathbf{x}') = \sum_{k,j} (f_k - f_j) \frac{\varphi_k(\mathbf{x})\varphi_j(\mathbf{x})\varphi_j(\mathbf{x}')\varphi_k(\mathbf{x}')}{(\epsilon_k - \epsilon_j)}, \tag{4}$$

where f_k and f_j are the occupation numbers of the KS states and we have assumed real orbitals. The interacting static linear response function χ is obtained from $\chi_{\rm S}$ and the Hartree-exchange-correlation kernel through the Dyson equation

$$\chi = \chi_{\rm S} + \chi_{\rm S} f_{\rm HXC} \chi. \tag{5}$$

Note that, as in the main text, when no frequency-dependence is indicated, the notation $f_{\rm HXC} = f_{\rm HXC}[n_0](\mathbf{r},\mathbf{r}') = \frac{\delta v_{\rm HXC}[n](\mathbf{r})}{\delta n(\mathbf{r}')}|_{n=n_0} = f_{\rm HXC}[n_0](\mathbf{r},\mathbf{r}',\omega = 0)$, the static limit of the frequency-dependent kernel; likewise for the response functions χ and $\chi_{\rm S}$. Pre-multiplying Eq. (5) by $\chi_{\rm S}^{-1}$ gives an expression for $\chi_{\rm S}^{-1}\chi$ that involves the interacting χ, so instead we formally solve for χ to obtain an expression directly in terms of KS quantities and the kernel:

$$\chi = \left(\mathbb{1} - f_{\rm HXC}\chi_{\rm S}\right)^{-1} \chi_{\rm S} \tag{6}$$

and hence

$$\chi_{\mathrm{S}}^{-1}\chi = \chi_{\mathrm{S}}^{-1}\left(\chi_{\mathrm{S}}^{-1} - f_{\mathrm{HXC}}\right)^{-1} = \left[\left(\chi_{\mathrm{S}}^{-1} - f_{\mathrm{HXC}}\right)\chi_{\mathrm{S}}\right]^{-1} = \left(\mathbb{1} - f_{\mathrm{HXC}}\chi_{\mathrm{S}}\right)^{-1}. \tag{7}$$

Including the spatial dependence, this equation reads

$$\int d^3x' \chi_{\mathrm{S}}^{-1}(\mathbf{x},\mathbf{x}')\chi(\mathbf{x}',\mathbf{r}) = \left(\mathbb{1} - f_{\mathrm{HXC}}\chi_{\mathrm{S}}\right)^{-1}(\mathbf{x},\mathbf{r}). \tag{8}$$

We turn now to the derivative of KS frequency $\nu_q = \epsilon_a - \epsilon_i$, where ϵ_a, ϵ_i are the orbital energies of the unoccupied and occupied KS orbitals involved in the qth KS excitation. By first-order (one-particle) perturbation theory, the variation of these energies under a variation of the KS potential is simply obtained from the expectation value of the variation v_{S} in these states:

$$\begin{aligned}\frac{\delta \nu_q}{\delta v_{\mathrm{S}}(\mathbf{x})} &= \frac{\delta}{\delta v_{\mathrm{S}}(\mathbf{x})}\left[\varepsilon_a - \varepsilon_i\right] = \frac{\delta}{\delta v_{\mathrm{S}}(\mathbf{x})}\left[\langle \phi_a | v_{\mathrm{S}} | \phi_a\rangle - \langle \phi_i | v_{\mathrm{S}} | \phi_i\rangle\right] \\ &= \int d^3x' \frac{\delta v_{\mathrm{S}}(\mathbf{x}')}{\delta v_{\mathrm{S}}(\mathbf{x})}\left[|\phi_a(\mathbf{x}')|^2 - |\phi_i(\mathbf{x}')|^2\right] \\ &= |\phi_a(\mathbf{x})|^2 - |\phi_i(\mathbf{x})|^2 = \Delta n_q^{\mathrm{KS}}(\mathbf{x})\end{aligned} \tag{9}$$

Utilizing Eq.(3) and Eq.(9), we write the first two derivatives in Eq.(2) as

$$\frac{\delta \nu_q^2}{\delta v_{\mathrm{ext}}(\mathbf{r})} = 2\nu_q \int d^3x \Delta n_q^{\mathrm{KS}}(\mathbf{x}) \left(\mathbb{1} - f_{\mathrm{HXC}}\chi_{\mathrm{S}}\right)^{-1}(\mathbf{x},\mathbf{r}) \tag{10}$$

$$\frac{\delta}{\delta v_{\mathrm{ext}}(\mathbf{r})}\sqrt{\nu_q \nu_{q'}} = \frac{1}{2}\int d^3x \left(\sqrt{\frac{\nu_{q'}}{\nu_q}}\Delta n_q^{\mathrm{KS}}(\mathbf{x}) + \sqrt{\frac{\nu_q}{\nu_{q'}}}\Delta n_{q'}^{\mathrm{KS}}(\mathbf{x})\right)\left(\mathbb{1} - f_{\mathrm{HXC}}\chi_{\mathrm{S}}\right)^{-1}(\mathbf{x},\mathbf{r}) \tag{11}$$

Now for the last term of Eq. 2, the derivative of the matrix-elements $f_{\mathrm{HXC},qq'}$ has contributions from derivatives of the KS orbitals, as well as through the density-dependence of the f_{HXC} kernel itself. Again, since we know how the orbitals change with v_{S} from first-order

perturbation theory, we will make use of the chain-rule of Eq. 3 and compute:

$$
\begin{aligned}
\frac{\delta f_{\rm HXC, qq'}[n_0](\omega)}{\delta v_{\rm S}(\mathbf{x})} &= \frac{\delta}{\delta v_{\rm S}(\mathbf{x})} \iint d^3x' d^3x'' \phi_i(\mathbf{x}') \phi_a(\mathbf{x}') f_{\rm HXC}(\mathbf{x}', \mathbf{x}'', \omega) \phi_j(\mathbf{x}'') \phi_b(\mathbf{x}'') \\
&= \iint d^3x' d^3x'' \frac{\delta \phi_i(\mathbf{x}')}{\delta v_{\rm S}(\mathbf{x})} \phi_a(\mathbf{x}') f_{\rm HXC}(\mathbf{x}', \mathbf{x}'', \omega) \phi_j(\mathbf{x}'') \phi_b(\mathbf{x}'') \\
&+ \iint d^3x' d^3x'' \phi_i(\mathbf{x}') \frac{\delta \phi_a(\mathbf{x}')}{\delta v_{\rm S}(\mathbf{x})} f_{\rm HXC}(\mathbf{x}', \mathbf{x}', \omega) \phi_j(\mathbf{x}'') \phi_b(\mathbf{x}'') \\
&+ \iint d^3x' d^3x'' \phi_i(\mathbf{x}') \phi_a(\mathbf{x}') f_{\rm HXC}(\mathbf{x}', \mathbf{x}'', \omega) \frac{\delta \phi_j(\mathbf{x}'')}{\delta v_{\rm S}(\mathbf{x})} \phi_b(\mathbf{x}'') \\
&+ \iint d^3x' d^3x'' \phi_i(\mathbf{x}') \phi_a(\mathbf{x}') f_{\rm HXC}(\mathbf{x}', \mathbf{x}'', \omega) \phi_j(\mathbf{x}'') \frac{\delta \phi_b(\mathbf{x}'')}{\delta v_{\rm S}(\mathbf{x})} \\
&+ \iint d^3x' d^3x'' \phi_i(\mathbf{x}') \phi_a(\mathbf{x}') \frac{\delta f_{\rm HXC}(\mathbf{x}', \mathbf{x}'', \omega)}{\delta v_{\rm S}(\mathbf{x})} \phi_j(\mathbf{x}'') \phi_b(\mathbf{x}''),
\end{aligned}
\tag{12}
$$

First-order (one-particle) perturbation theory directly gives the orbital derivative

$$
\frac{\delta \phi_i(\mathbf{x}')}{\delta v_{\rm S}(\mathbf{x})} = \sum_{p \neq i}^{\infty} \frac{\phi_p(\mathbf{x}) \phi_p(\mathbf{x}')}{\epsilon_i - \epsilon_p} \phi_i(\mathbf{x}) = G_{\rm S}(\mathbf{x}', \mathbf{x}) \phi_i(\mathbf{x}). \tag{13}
$$

where we have defined the KS Green's function, $G_{\rm S}(\mathbf{x}', \mathbf{x})$. This allows us to replace the double-integrals in Eq. (12) by various off-diagonal matrix elements of the $f_{\rm HXC}$ kernel, multiplied by products of two KS orbitals (see shortly). For the last term of Eq.(12), it is more direct to compute directly the needed derivative with respect to $v_{\rm ext}$, as follows:

$$
\frac{\delta f_{\rm HXC}[n_0](\mathbf{x}', \mathbf{x}'', \omega)}{\delta v_{\rm ext}(\mathbf{r})} = \int d^3x \left. \frac{\delta f_{\rm HXC}[n_0'](\mathbf{x}', \mathbf{x}'', \omega)}{\delta n_0'(\mathbf{x})} \right|_{n_0'=n_0} \chi[n_0](\mathbf{x}, \mathbf{r}), \tag{14}
$$

where n_0' denotes a general ground-state density. Since $f_{\rm H}(\mathbf{x}, \mathbf{x}') = 1/|\mathbf{x} - \mathbf{x}'|$, it has no density-dependence, and we define the kernel appearing here as

$$
\tilde{g}_{\rm XC}[n_0](\mathbf{x}', \mathbf{x}'', \mathbf{x}, \omega) = \left. \frac{\delta f_{\rm XC}[n_0'](\mathbf{x}', \mathbf{x}'', \omega)}{\delta n_0'(\mathbf{x})} \right|_{n_0'=n_0} \tag{15}
$$

In general, this is distinct from the second-order response kernel, $g_{\rm XC}[n_0](\mathbf{x}, \mathbf{x}', \mathbf{x}'', \omega, \omega')$ which is the Fourier transform of $g_{\rm XC}[n_0](\mathbf{x}, \mathbf{x}', \mathbf{x}'', t-t', t-t'') = \left. \frac{\delta^2 v_{\rm XC}[n](\mathbf{r},t)}{\delta n(\mathbf{r}',t') \delta n(\mathbf{r}'',t'')} \right|_{n=n_0}$. However,

with an adiabatic approximation, $v_{\mathrm{XC}}^{\mathrm{adia}}[n](\mathbf{r},t) = \delta E_{\mathrm{XC}}[n]/\delta n(\mathbf{r},t)$, and the kernels become frequency-independent and no longer distinct, $\tilde{g}_{\mathrm{XC}}^{\mathrm{adia}}[n_0](\mathbf{x},\mathbf{x}',\mathbf{x}'') = g_{\mathrm{XC}}^{\mathrm{adia}}[n_0](\mathbf{x},\mathbf{x}',\mathbf{x}'')$.

Putting Eqs. 9– 15 together into Eq. 2, we finally obtain

$$\begin{aligned}\frac{\delta\Omega_{qq'}(\omega)}{\delta v_{\mathrm{ext}}\,(r)} = \int d^3x \Bigg\{ & 2\nu_q \Delta n_q^{\mathrm{KS}}(\mathbf{x})\delta_{qq'} + 2f_{\mathrm{HXC},qq'}\,(\omega)\left(\sqrt{\frac{\nu_{q'}}{\nu_q}}\Delta n_q^{\mathrm{KS}}(\mathbf{x}) + \sqrt{\frac{\nu_q}{\nu_{q'}}}\Delta n_{q'}^{\mathrm{KS}}(\mathbf{x})\right) \\ & + 4\sqrt{\nu_q\nu_{q'}}\Bigg(\sum_{k\neq i}^{\infty}\frac{1}{\epsilon_i-\epsilon_k} f_{\mathrm{HXC},\,ka,\,jb}\,(\omega)\,\Phi_{ik}(\mathbf{x}) + \sum_{k\neq a}^{\infty}\frac{1}{\epsilon_a-\epsilon_k} f_{\mathrm{HXC},\,ik,\,jb}\,(\omega)\,\Phi_{ka}(\mathbf{x}) \\ & + \sum_{k\neq j}^{\infty}\frac{1}{\epsilon_j-\epsilon_k} f_{\mathrm{HXC},\,ia,\,kb}\,(\omega)\,\Phi_{jk}(\mathbf{x}) + \sum_{k\neq b}^{\infty}\frac{1}{\epsilon_b-\epsilon_k} f_{\mathrm{HXC},\,ia,\,jk}\,(\omega)\,\Phi_{kb}(\mathbf{x})\Bigg)\Bigg\}\left(\mathbb{1} - f_{\mathrm{HXC}}\chi_{\mathrm{S}}\right)^{-1}(\mathbf{x},\mathbf{r}) \\ & + 4\sqrt{\nu_q\nu_{q'}}\int d^3x\,\tilde{g}_{\mathrm{XC},qq'}\,(\mathbf{x},\omega)\,\chi(\mathbf{x},\mathbf{r})\end{aligned} \tag{16}$$

2 Single-Transition Limit

While the SMA expression for the excited state density difference includes only TDDFT diagonal correction and neglects the f_{XC}-coupling to the other excitations, these other excitations do enter into the functional derivative involved in obtaining the excited-state density in the form of KS transition densities through the $\left(\mathbb{1} - f_{\mathrm{HXC}}\chi_{\mathrm{S}}\right)^{-1}$ and response χ that appear in the last two terms of Eq. (21) of the main text. In the single-transition limit (STL), the system is assumed to have only one KS excitation, the one underlying the excitation of interest I. In that case, *all* quantities involved in the density-difference expression are evaluated with only two KS orbitals. Here we provide the compact expression for the density difference $\Delta n_I^{\mathrm{KS}}(\mathbf{r})$ in the STL, with the correction to the KS density difference explicitly given by a single KS transition density $\Phi_q(\mathbf{r})$. We further make a connection between our approach and the result of Ref.[S1] under the STL.

We begin by including only $p = i, a$ in the sums in Eq. (21), which gives

$$\begin{aligned}\Delta n_I(r) =& \frac{G_I^2}{\omega_I} \int d^3x \left\{ \left[\left(\nu_q + 2 f_{\text{HXC},qq}(\omega) \right) \Delta n_q^{\text{KS}}(\mathbf{r}) \right.\right. \\ & + 4 \left(f_{\text{Hxc},\,ii,\,ia}(\omega) - f_{\text{Hxc},\,aa,\,ia}(\omega) \right) \Phi_{ia}(\mathbf{x}) \big] \left(\mathbb{1} - f_{\text{HXC}}(0) \chi_{\text{S}} \right)^{-1} (\mathbf{x}, \mathbf{r}) \\ & \left. + 2 \nu_q \tilde{g}_{\text{xc},\,ia,\,ia}(\mathbf{x}, \omega) \chi(\mathbf{x}, \mathbf{r}) \right\},\end{aligned} \tag{17}$$

As discussed in the main text, we expand $\left(\mathbb{1} - f_{\text{HXC}} \chi_{\text{S}} \right)^{-1}$ and χ in first-order of f_{HXC}, e.g.

$$\left(\mathbb{1} - f_{\text{HXC}} \chi_{\text{S}} \right)^{-1} (\mathbf{x}, \mathbf{r}) \approx \delta(\mathbf{x} - \mathbf{r}) + \int d^3x' f_{\text{HXC}}(\mathbf{x}, \mathbf{x}') \chi_{\text{S}}(\mathbf{x}', \mathbf{r}) \,. \tag{18}$$

Interestingly, in the case of the truncation to one single transition, the Taylor series expansion can be exactly resummed. To see this, we note that in this case, the KS linear response takes the form $\chi_{\text{S}}(\mathbf{x}', \mathbf{x}'') = 4 \frac{\Phi_q(\mathbf{x}') \Phi_q(\mathbf{x}'')}{-\nu_q}$, so, going to higher-order in the Taylor series gives

$$\begin{aligned}\left(\mathbb{1} - f_{\text{HXC}} \chi_{\text{S}} \right)^{-1} (\mathbf{x}, \mathbf{r}) =& \delta(\mathbf{x} - \mathbf{r}) + 4 \int d^3x' f_{\text{HXC}}(\mathbf{x}, \mathbf{x}') \frac{\Phi_q(\mathbf{x}') \Phi_q(\mathbf{r})}{-\nu_q} \\ & + 4^2 \iiint d^3x' d^3x'' d^3x''' f_{\text{HXC}}(\mathbf{x}, \mathbf{x}') \frac{\Phi_q(\mathbf{x}') \Phi_q(\mathbf{x}'')}{-\nu_q} f_{\text{HXC}}(\mathbf{x}'', \mathbf{x}''') \frac{\Phi_q(\mathbf{x}''') \Phi_q(\mathbf{r})}{-\nu_q} \\ & + \ldots\end{aligned} \tag{19}$$

Each term in the expansion, except for the zeroth order, is $(\mathbf{x}, \mathbf{r})$-dependent through the common multiplier $-\frac{4}{\nu_q} f_{\text{HXC},\,q}(\mathbf{x}) \Phi_q(\mathbf{r})$ where we defined $f_{\text{HXC},\,q}(\mathbf{x}) = \int d^3x' f_{\text{HXC}}(\mathbf{x}, \mathbf{x}') \Phi_q(\mathbf{x}')$ to take care of the integral over $\mathbf{x}'$. Starting with the third term, each new order of $f_{\text{HXC}} \chi_{\text{S}}$ integral brings an additional term $-\frac{4 f_{\text{HXC},qq}}{\nu_q}$. Thus we can resum the right-hand-side of

Eq.(19):

$$\begin{aligned}\left(\mathbb{1}-f_{\text{HXC}}\chi_{\text{S}}\right)^{-1}(\mathbf{x},\mathbf{r}) =&\delta(\mathbf{x}-\mathbf{r})-\frac{4}{\nu_q}f_{\text{HXC},\,q}(\mathbf{x})\Phi_q(\mathbf{r})\left(1-\frac{4f_{\text{HXC},qq}}{\nu_q}+\left(\frac{4f_{\text{HXC},qq}}{\nu_q}\right)^2-\left(\frac{4f_{\text{HXC},qq}}{\nu_q}\right)^3+\dots\right)\\ =&\delta(\mathbf{x}-\mathbf{r})-\frac{4}{\nu_q}f_{\text{HXC},\,q}(\mathbf{x})\Phi_q(\mathbf{r})\frac{1}{1+\frac{4f_{\text{HXC},qq}}{\nu_q}}\\ =&\delta(\mathbf{x}-\mathbf{r})-\frac{4}{\nu_q+4f_{\text{HXC},qq}}f_{\text{HXC},\,q}(\mathbf{x})\Phi_q(\mathbf{r})\end{aligned} \tag{20}$$

Although not needed for present purposes, we note in passing that this is also true for the finite-frequency case in the STL limit:

$$\left(\mathbb{1}-f_{\text{HXC}}(\omega)\chi_{\text{S}}(\omega)\right)^{-1}(\mathbf{x},\mathbf{r})=\delta(\mathbf{x}-\mathbf{r})+\frac{4}{\omega-(\nu_q+4f_{\text{HXC},qq})}f_{\text{HXC},\,q}(\mathbf{x},\omega)\Phi_q(\mathbf{r})\,. \tag{21}$$

Likewise, a similar exact resumming can be done for the interacting response function χ defined with the Dyson equation:

$$\begin{aligned}\chi(\mathbf{x},\mathbf{r}) =&\chi_{\text{S}}(\mathbf{x},\mathbf{r})+\iint d^3x'd^3x''\chi_{\text{S}}(\mathbf{x},\mathbf{x}')f_{\text{HXC}}(\mathbf{x}',\mathbf{x}'')\chi_{\text{S}}(\mathbf{x}'',\mathbf{r})\\ &+\iiiint d^3x'd^3x''d^3x'''d^3x^{\text{iv}}\chi_{\text{S}}(\mathbf{x},\mathbf{x}')f_{\text{HXC}}(\mathbf{x}',\mathbf{x}'')\chi_{\text{S}}(\mathbf{x}'',\mathbf{x}''')f_{\text{HXC}}(\mathbf{x}''',\mathbf{x}^{\text{iv}})\chi_{\text{S}}(\mathbf{x}^{\text{iv}},\mathbf{r})+\dots\end{aligned} \tag{22}$$

Utilizing again the KS response function in the two-state limit, one may extract a common $(\mathbf{x},\mathbf{r})$-dependent multiplier and resum, finally obtaining

$$\chi(\mathbf{x},\mathbf{r})=\frac{\nu_q}{\nu_q+4f_{\text{HXC},qq}}\chi_{\text{S}}(\mathbf{x},\mathbf{r})\,. \tag{23}$$

And again, although not needed here, we note the resummation holds also for finite frequencies within STL:

$$\chi(\mathbf{x},\mathbf{r},\omega)=\frac{\omega-\nu_q}{\omega-(\nu_q+4f_{\text{HXC},qq})}\chi_{\text{S}}(\mathbf{x},\mathbf{r},\omega)\,. \tag{24}$$

Here we recognize the factor from the oscillator strength sum-rule for the single-transition case.

Returning now to Eq.(17), and inserting Eq.(20) and Eq.(23), we find the excited-state density difference:

$$\begin{aligned}\Delta n_I^{\rm STL}(\mathbf{r}) =& \frac{G_I^2}{\omega_I}\left\{\left(\nu_q + 2 f_{\rm HXC,\mathit{qq}}(\omega_I)\right)\Delta n^{\rm KS}(\mathbf{r})\right.\\ &+ \frac{8}{\nu_q + 4 f_{\rm HXC,\mathit{qq}}(0)}\left[\left(\nu_q + f_{\rm HXC,\mathit{qq}}(\omega_I)\right)\left(f_{\mathrm{HXC},\,ii,\,ia}(\omega_I) - f_{\mathrm{HXC},\,aa,\,ia}(\omega_I)\right)\right.\\ &- \left.\left.\nu_q \tilde{g}_{\rm XC,\mathit{qqq}}(\omega_I)\right]\Phi_q(\mathbf{r})\right\},\end{aligned} \tag{25}$$

2.1 Equivalence to Furche and Ahlrichs Approach

Here we show explicitly that our formula in the STL limit derived from the real-space approach agrees with the variational approach in the density-matrix picture in the seminal work of Furche.[S1,S2]

Ref.[S1,S2] define the difference density matrix P in the KS orbital basis (i occupied, a unoccupied, σ denotes spin):

$$P_{ia\sigma} = T_{ia\sigma} + Z_{ia\sigma}, \tag{26}$$

where the matrix T is the so-called unrelaxed difference density matrix defined as

$$T_{ia\sigma} = T_{ai\sigma} = 0 \tag{27}$$

$$T_{bc\sigma} = \frac{1}{2}\sum_j \left\{(X+Y)_{jb\sigma}(X+Y)_{jc\sigma} + (X-Y)_{jb\sigma}(X-Y)_{jc\sigma}\right\} \tag{28}$$

$$T_{jk\sigma} = -\frac{1}{2}\sum_b \left\{(X+Y)_{jb\sigma}(X+Y)_{kb\sigma} + (X-Y)_{jb\sigma}(X-Y)_{kb\sigma}\right\}. \tag{29}$$

Here, X and Y are vectors indexed by single excitations and deexcitations representing the transition densities obtained from linear-response TDDFT. The Z-vector of Eq. (26) is defined through

$$\sum_{j,b,\sigma'} (A+B)_{ia\sigma,\,jb\sigma'} Z_{jb\sigma'} = -R_{ia\sigma}, \tag{30}$$

where R is

$$R_{ia\sigma} = 2\sum_{b}(X+Y)_{ib\sigma}\sum_{rs\sigma'} f_{\text{HXC}ab\sigma,\,rs\sigma'}(X+Y)_{rs\sigma} - 2\sum_{j}(X+Y)_{ja\sigma}\sum_{rs\sigma'} f_{\text{HXC}ji\sigma,\,rs\sigma'}(X+Y)_{rs\sigma} + 2\sum_{rs\sigma'} f_{\text{HXC}ia\sigma,\,rs\sigma'}T_{rs\sigma} + 2\sum_{jb\sigma'\,kc\sigma''} g_{\text{XC}ia\sigma,\,jb\sigma',\,kc\sigma''}(X+Y)_{jb\sigma'}(X+Y)_{kc\sigma''} \tag{31}$$

and $A_{ia\sigma,\,jb\sigma'} = \nu_{ia\sigma}\delta_{ij}\delta_{ab}\delta_{\sigma\sigma'} + 2f_{\text{HXC}ia\sigma,\,jb\sigma'}$, $B_{ia\sigma,\,jb\sigma'} = 2f_{\text{HXC}ia\sigma,\,jb\sigma'}$. In establishing Eqs. (30)–(31), the authors implicitly assumed the adiabatic approximation in the f_{XC} kernel and in their normalization of the X and Y vectors.

Now, in the STL, the transition vectors reduce to[S2]

$$(X+Y)_{jb\sigma} = \sqrt{\frac{\nu_{ia\sigma}}{2\omega_I}}\delta_{ij}\delta_{ab} \quad \text{and} \quad (X-Y)_{jb\sigma} = \sqrt{\frac{\omega_I}{2\nu_{ia\sigma}}}\delta_{ij}\delta_{ab}, \tag{32}$$

where $\omega_I^2 = \nu_{ia\sigma}^2 + 2\sum_{\sigma'}\sqrt{\nu_{ia\sigma}\nu_{ia\sigma'}}f_{\text{HXC}ia\sigma,ia\sigma'}$. We can further simplify the unrelaxed density difference Eq. (28) and Eq. (29):

$$T_{bc\sigma} = \frac{1}{4}\frac{\nu_{ia\sigma}^2 + \omega_I^2}{\nu_{ia\sigma}\omega_I}\delta_{ab}\delta_{ac}, \quad T_{jk\sigma} = -\frac{1}{4}\frac{\nu_{ia\sigma}^2 + \omega_I^2}{\nu_{ia\sigma}\omega_I}\delta_{ij}\delta_{ik}. \tag{33}$$

The Z-vector Eq. (30) and R in Eq. (31) reduce to

$$Z_{ia\sigma} = \frac{R_{ia\sigma}}{\sum_{\sigma'}\nu_{ia\sigma}\delta_{\sigma\sigma'} + 2f_{\text{HXC}ia\sigma,\,ia\sigma'}} \tag{34}$$

and

$$R_{ia\sigma} = \frac{\nu_{ia\sigma}}{\omega_I}\sum_{\sigma'}\left(f_{\text{HXC}aa\sigma,\,ia\sigma'} - f_{\text{HXC}ii\sigma,\,ia\sigma'}\right) + \frac{1}{2}\frac{\nu_{ia\sigma}^2 + \omega_I^2}{\nu_{ia\sigma}\omega_I}\left(f_{\text{HXC}ia\sigma,\,aa\sigma'} - f_{\text{HXC}ia\sigma,\,ii\sigma'}\right) + \sum_{\sigma'\sigma''}\frac{\sqrt{\nu_{ia\sigma'}\nu_{ia\sigma''}}}{\omega_I}g_{\text{XC}ia\sigma,\,ia\sigma',\,ia\sigma''} \tag{35}$$

For the case we are interested in, we write all quantities as spin-unpolarized: $\omega_I^2 =$

$\nu_{ia}^2 + 4\nu_{ia} f_{\text{HXC}ia,\,ia}$, and

$$Z_{ia} = \frac{1}{\nu_{ia} + 4 f_{\text{HXC}ia,\,ia}} \left\{ 8 \frac{\nu_{ia} + f_{\text{HXC}ia,\,ia}}{\sqrt{\nu_{ia}^2 + 4\nu_{ia} f_{\text{HXC}ia,\,ia}}} \left(f_{\text{HXC}ii,\,ia} - f_{\text{HXC}aa,\,ia} \right) - 8 \frac{\nu_{ia}}{\sqrt{\nu_{ia}^2 + 4\nu_{ia} f_{\text{HXC}ia,\,ia}}} g_{\text{XC}ia,\,ia,\,ia} \right\} \tag{36}$$

$$T_{aa} = T_{aa\uparrow} + T_{aa\downarrow} = \frac{\nu_{ia} + 2 f_{\text{HXC}ia,\,ia}}{\omega_I}, \quad T_{ii} = T_{ii\uparrow} + T_{ii\downarrow} = -\frac{\nu_{ia} + 2 f_{\text{HXC}ia,\,ia}}{\omega_I} \tag{37}$$

Finally, we insert the Eqs. (36–37) into Eq. (26), and obtain the real-space representation of the density difference from the difference density matrix P as $\Delta n_I(\mathbf{r}) = P(\mathbf{r}, \mathbf{r})$, where P expressed in the real-space as $P(\mathbf{r}, \mathbf{r}') = \sum_{jk} \phi_j(\mathbf{r}) P_{jk} \phi_k(\mathbf{r}')$. The expression for the density difference in the single-transition limit is then

$$\begin{aligned} \Delta n_I(\mathbf{r}) = & \frac{1}{\omega_I} \left\{ \left(\nu_q + 2 f_{\text{HXC},qq} \right) \Delta n^{\text{KS}}(\mathbf{r}) \right. \\ & \left. + \frac{8}{\nu_q + 4 f_{\text{HXC},qq}} \left[\left(\nu_q + f_{\text{HXC},qq} \right) \left(f_{\text{HXC}ii,\,ia} - f_{\text{HXC}aa,\,ia} \right) - \nu_q g_{\text{XC},qqq} \right] \Phi_q(\mathbf{r}) \right\}. \end{aligned} \tag{38}$$

This result agrees with Eq. (25) here and Eq. (25) of the main text, but only within adiabatic approximation, where $G_I^2 = 1$ and f_{HXC} is taken at $\omega = 0$. We note that the approach of Furche and Ahlrichs does not apply in the non-adiabatic regime.

3 DSMA: Derivatives of the Hamiltonian Matrix Elements

To evaluate the density-difference for states of double-excitation character using DSMA and DSPA, we need the functional derivatives with respect to the external potential of the Hamiltonian matrix elements that appear in those expressions. For the DSMA/DSPA variant we have considered, these are the derivatives of H_{qD}, H_{DD}, H_{00} where the Hamiltonian $H = T + V_{\text{ext}} + W = \sum_i h_i + \sum_{i \neq j} w_{ij}$, a sum of one-body and two-body terms.

The states $q, D, 0$ are Slater determinants or a sum of two Slater determinants (spin-

adapted), and so to evaluate the matrix elements we can use the Slater-Condon rules, and then take the functional derivative. The Slater-Condon rules[S3–S5] enable us to reduce these to the computation of a few one and two electron integrals.

For the one-body terms, we need:

$$\begin{aligned}\frac{\delta}{\delta v_{\rm ext}(\mathbf{r})}\langle\phi_r|\hat{h}|\phi_s\rangle &= \int d^3x \frac{\delta\phi_r(\mathbf{x})}{\delta v_{\rm ext}(\mathbf{r})}h(\mathbf{x})\phi_s(\mathbf{x}) + \int d^3x\phi_r(\mathbf{x})h(\mathbf{x})\frac{\delta\phi_s(\mathbf{x})}{\delta v_{\rm ext}(\mathbf{r})} \\ &+ \int d^3x\phi_r(\mathbf{x})\frac{\delta h(\mathbf{x})}{\delta v_{\rm ext}(\mathbf{r})}\phi_s(\mathbf{x}),\end{aligned} \tag{39}$$

written here assuming real orbitals but easily generalizable to complex orbitals. The derivatives in the first two terms can be evaluated by applying the chain rule from Eq. (3) and expanding the derivative of orbital with respect to $v_{\rm S}$ using the Green's function definition (Eq. (13)), while the last term can be simply reduced to

$$\int d^3x\phi_r(\mathbf{x})\frac{\delta h(\mathbf{x})}{\delta v_{\rm ext}(\mathbf{r})}\phi_s(\mathbf{x}) = \int d^3x\phi_r(\mathbf{x})\frac{\delta v_{\rm ext}(\mathbf{x})}{\delta v_{\rm ext}(\mathbf{r})}\phi_s(\mathbf{x}) = \Phi_{rs}(\mathbf{r}). \tag{40}$$

using the definition from earlier, $\Phi_{rs}(\mathbf{r}) = \phi_r(\mathbf{r})\phi_s(\mathbf{r})$.

For the integral of the two-body integrals we need:

$$\begin{aligned}\frac{\delta}{\delta v_{\rm ext}(\mathbf{r})}(\phi_r\phi_s|\phi_m\phi_n) &= \iint d^3xd^3x'\frac{\delta\phi_r(\mathbf{x})}{\delta v_{\rm ext}(\mathbf{r})}\phi_s(\mathbf{x}')w(\mathbf{x},\mathbf{x}')\phi_m(\mathbf{x}')\phi_n(\mathbf{x}') \\ &+ \iint d^3xd^3x'\phi_r(\mathbf{x})\frac{\delta\phi_s(\mathbf{x}')}{\delta v_{\rm ext}(\mathbf{r})}w(\mathbf{x},\mathbf{x}')\phi_m(\mathbf{x}')\phi_n(\mathbf{x}') \\ &+ \iint d^3xd^3x'\phi_r(\mathbf{x})\phi_s(\mathbf{x}')w(\mathbf{x},\mathbf{x}')\frac{\delta\phi_m(\mathbf{x}')}{\delta v_{\rm ext}(\mathbf{r})}\phi_n(\mathbf{x}') \\ &+ \iint d^3xd^3x'\phi_r(\mathbf{x})\phi_s(\mathbf{x}')w(\mathbf{x},\mathbf{x}')\phi_m(\mathbf{x}')\frac{\delta\phi_n(\mathbf{x}')}{\delta v_{\rm ext}(\mathbf{r})}\end{aligned} \tag{41}$$

where the derivatives of the orbitals are evaluated in the same way.

After expanding the derivatives of the KS orbitals, we may simplify the resulting one-

and two-body integrals, analogously to what we did for Eq. (12). This finally yields

$$\frac{\delta}{\delta v_{\text{ext}}(\mathbf{r})}\langle\phi_r|\hat{h}|\phi_s\rangle = \Phi_{rs}(\mathbf{r}) + \int d^3x \left\{\sum_{p\neq r}^{\infty}\frac{\langle\phi_p|\hat{h}|\phi_s\rangle}{\epsilon_r-\epsilon_p}\Phi_{pr}(\mathbf{x}) + \sum_{p\neq s}^{\infty}\frac{\langle\phi_r|\hat{h}|\phi_p\rangle}{\epsilon_s-\epsilon_p}\Phi_{ps}(\mathbf{x})\right\}\left(\mathbb{1}-f_{\text{HXC}}(0)\chi_{\text{S}}\right)^{-1}(\mathbf{x},\mathbf{r}), \tag{42}$$

$$\frac{\delta}{\delta v_{\text{ext}}(\mathbf{r})}(\phi_r\phi_s|\phi_m\phi_n) = \int d^3x \left\{\sum_{p\neq r}^{\infty}\frac{(\phi_p\phi_s|\phi_m\phi_n)}{\epsilon_r-\epsilon_p}\Phi_{pr}(\mathbf{x}) + \sum_{p\neq s}^{\infty}\frac{(\phi_r\phi_p|\phi_m\phi_n)}{\epsilon_s-\epsilon_p}\Phi_{ps}(\mathbf{x}) + \sum_{p\neq m}^{\infty}\frac{(\phi_r\phi_s|\phi_p\phi_n)}{\epsilon_m-\epsilon_p}\Phi_{pm}(\mathbf{x}) + \sum_{p\neq n}^{\infty}\frac{(\phi_r\phi_s|\phi_m\phi_p)}{\epsilon_n-\epsilon_p}\Phi_{pn}(\mathbf{x})\right\}\left(\mathbb{1}-f_{\text{HXC}}(0)\chi_{\text{S}}\right)(\mathbf{x},\mathbf{r}), \tag{43}$$

which are the ingredients for the derivatives of the matrix elements H_{ij}.

References

(S1) Furche, F.; Ahlrichs, R. Adiabatic time-dependent density functional methods for excited state properties. *The Journal of Chemical Physics* **2002**, *117*, 7433–7447.

(S2) Furche, F. On the density matrix based approach to time-dependent density functional response theory. *The Journal of Chemical Physics* **2001**, *114*, 5982–5992.

(S3) Slater, J. C. The Theory of Complex Spectra. *Physical Review* **1929**, *34*, 1293–1322, Publisher: American Physical Society.

(S4) Condon, E. U. The Theory of Complex Spectra. *Physical Review* **1930**, *36*, 1121–1133, Publisher: American Physical Society.

(S5) Szabo, A. *Modern quantum chemistry: introduction to advanced electronic structure theory*; Dover Publications: Mineola, N.Y, 1996.
